# Interior Decorative VOCs Elevate T Cell‐Mediated Obstructive Lung Disease Risks via Osteogenesis‐Driven Lymphoid‐Biased Hematopoiesis

**DOI:** 10.1002/advs.202512663

**Published:** 2025-11-05

**Authors:** Hongyan Yu, Jingxu Zhang, Qingping Liu, Duxing Li, Ruonan Pan, Gan Miao, Yidi Chen, Zhe Kou, Guangbo Qu, Rong Zhang, Xiaoting Jin, Yuxin Zheng

**Affiliations:** ^1^ Department of Occupational and Environmental Health School of Public Health Qingdao University Qingdao Shandong 266071 China; ^2^ Department of Toxicology School of Public Health Hebei Medical University Shijiazhuang Hebei 050017 China; ^3^ ZaoZhuang Center for Disease Control and Prevention Zaozhuang Shandong 277101 China; ^4^ State Key Laboratory of Environmental Chemistry and Ecotoxicology Research Center for Eco‐Environmental Sciences Chinese Academy of Sciences Beijing 100085 China; ^5^ College of Resources and Environment University of Chinese Academy of Sciences (UCAS) Beijing 101408 China; ^6^ School of Environment Hangzhou Institute for Advanced Study, UCAS Hangzhou Zhejiang 310058 China

**Keywords:** volatile organic compounds, T cells, lymphoid‐biased hematopoiesis, osteogenic differentiation, obstructive lung diseases

## Abstract

Volatile organic compounds (VOCs) emitted from interior decorations are suggested to pose substantial respiratory hazards as they disturb the immune response in the lung. Despite this, the characteristics and mechanisms underlying immune cell responses to real‐world VOC mixtures, rather than previously individual or dominant VOC species, remain poorly understood. Employing a whole‐body inhalation exposure model, we reveal that interior decorative VOCs alter pulmonary immune cell profiles with a persistent increase in alveolar T cells. This sustained elevation results from intensified lymphoid‐biased hematopoiesis in the bone marrow (BM) rather than in situ lung, which is followed by thymic maturation and subsequent recruitment to lung tissues. The ex vivo biosensor and antibody neutralization assays clarify that VOCs‐induced lymphoid‐biased hematopoiesis is primarily driven by osteogenic differentiation within the BM niche, a process further regulated by interleukin‐6 (IL‐6) and interleukin‐17A (IL‐17A). Moreover, the cohort study associates VOCs‐expanded lymphocytes with increased risks of obstructive lung diseases, where exposed individuals show elevated IL‐6 and IL‐17A levels, correlating with VOC concentrations and lymphocyte proportions. These findings highlight compelling indicators (i.e., T cells) and potential interventions (i.e., IL‐6 and IL‐17A) for evaluating and mitigating VOC‐associated respiratory risks.

## Graphical abstract

This study utilizes a whole‐body inhalation model to reveal that interior decorative VOCs exposure persistently elevates alveolar T cells. This increase is attributed to lymphoid‐biased hematopoiesis in the bone marrow, which is further driven by IL‐6/IL‐17A‐mediated osteogenic differentiation. A population‐based cohort links VOCs‐induced lymphocyte expansion to obstructive lung disease risk, highlighting broader public health implications.

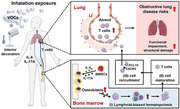

## 1 Introduction

Air pollution accounts for 6.7 million annual deaths globally, with indoor air pollution responsible for nearly half of this burden,^[^ ^1^ ^]^ highlighting the severity of this issue. Volatile organic compounds (VOCs), one of the typical indoor pollutants, are extensively detected in homes at levels often a staggering ten times higher than outdoors,^[^ ^2^ ^]^ particularly considering that humans spend over 90% of their time indoors.^[^ ^3^ ^]^ Epidemiological studies consistently linked VOCs to respiratory diseases,^[^ ^4^, ^5^ ^]^ yet the mechanisms underlying this association remain elusive. In vivo studies demonstrated that certain VOC components, such as formaldehyde, benzene, and toluene, can trigger severe lung diseases, which are believed to be caused by immune disruption, inflammation, oxidative stress, etc.^[^ ^6^, ^7^ ^]^ However, limited information is available on the toxicity of environmental VOC mixtures, which encompass over 300 distinct chemical species.^[^ ^8^ ^]^ This knowledge gap stems partly from the lack of suitable exposure models that accurately reflect the complexity of real‐world VOC mixtures.

An upward trend in indoor VOC levels, largely attributed to the prevalence of interior decoration,^[^ ^9^ ^]^ is a major contributor to indoor air pollution. To delineate the toxicological implications of interior decorative VOCs, we have established a whole‐body inhalation exposure model to simulate real‐life interior decorative exposure scenarios.^[^ ^10^ ^]^ Using this non‐invasive model, we identified 116 distinct VOC species,^[^ ^10^ ^]^ providing an ideal platform to investigate the toxic effects and underlying mechanisms of real‐life interior VOCs exposure. Moreover, this model allows for in‐depth studies under controllable VOC concentrations. The VOC concentrations in the control group of our study were rigorously maintained below the permissible limit,^[^ ^11^ ^]^ while the exposure group was designed to mimic the first year following interior decoration.^[^ ^12^ ^]^ Our prior study has discovered that interior decorative VOCs can induce excessive inflammation that contributes to the progression of disease,^[^ ^10^ ^]^ highlighting their potential to disrupt immune response. Despite this, the impact and underlying mechanisms of VOCs exposure on the immune response remain less understood.

Due to their foundational role in the immune response to inhalation stimuli, immune cell composition in the lung has garnered significant public interest.^[^ ^13^ ^]^ Exposure to individual VOC components, such as formaldehyde, can aggravate the infiltration of neutrophils (NEs) into the lung,^[^ ^14^ ^]^ which is a hallmark of inflammatory lung injury. Major VOC components have been shown to cause the accumulation of macrophages (Macros), NEs, and lymphocytes (LYM) within the alveoli.^[^ ^15^ ^]^ In the lung microenvironment, the alveoli serves as a primary line of defense against harmful agents,^[^ ^16^ ^]^ while the lung interstitium (LI) supports structural integrity.^[^ ^17^ ^]^ The distinct anatomical separation between the alveoli and LI assigns unique functional roles to their immune cells, acting as predictive markers for assessing specific lung diseases.^[^ ^18^, ^19^ ^]^ One of the critical unresolved questions is to clarify the effects of VOCs on the compositions of immune cells within the lung microenvironment, including both alveoli and LI, with the intent to identify compelling indicators of the lung risks posed by VOCs exposure.

As a pivotal regulator of immune cell composition, hematopoiesis is organized in a hierarchical and demand‐driven manner, ensuring a continuous supply of immune cells.^[^ ^20^ ^]^ Major VOC components, such as formaldehyde and benzene, have been identified as hematopoiesis‐stimulating agents,^[^ ^21^, ^22^ ^]^ indicating their stimulatory effects on hematopoiesis. In addition to the bone marrow (BM), extramedullary organs, including the liver, spleen, and lung, also possess hematopoietic reconstitution capacity,^[^ ^23^, ^24^ ^]^ with their responsiveness dependent on the exposure route. Notably, extramedullary hematopoiesis in the liver and spleen is primarily activated by oral exposure to environmental stimuli,^[^ ^24^ ^]^ while pulmonary hematopoiesis is more responsive to inhalational stimuli. In our previous study, fine particulate matter strongly activated myeloid‐biased hematopoiesis in the lung,^[^ ^25^ ^]^ underscoring its susceptibility to inhaled toxicants. Thus, a comprehensive investigation is necessitated to elucidate the contribution of both in situ pulmonary and distal BM hematopoiesis to immune cell replenishment in lung tissues under VOCs exposure, as well as to delve into the underlying mechanisms.

In the present study, we sought to identify the characteristics of pulmonary immune cell compositions, elucidate the underlying mechanisms, and reveal the detrimental respiratory impacts induced by interior decorative VOCs exposure. For these aims, we established a whole‐body inhalation VOCs exposure model, conducted in vivo mouse experiments, ex vivo analyses, and a population‐based cohort study. Our work not only identifies T cells as a critical indicator for assessing the lung risks associated with environmental VOCs, but also provides novel insights into the mechanisms by which environmental pollutants disrupt immune cell compositions from the perspective of immune cell renewal.

## 2 Results

### 2.1 Alteration of Immune Cell Composition in the Lung Stimulated by VOCs

**Figure 1:**
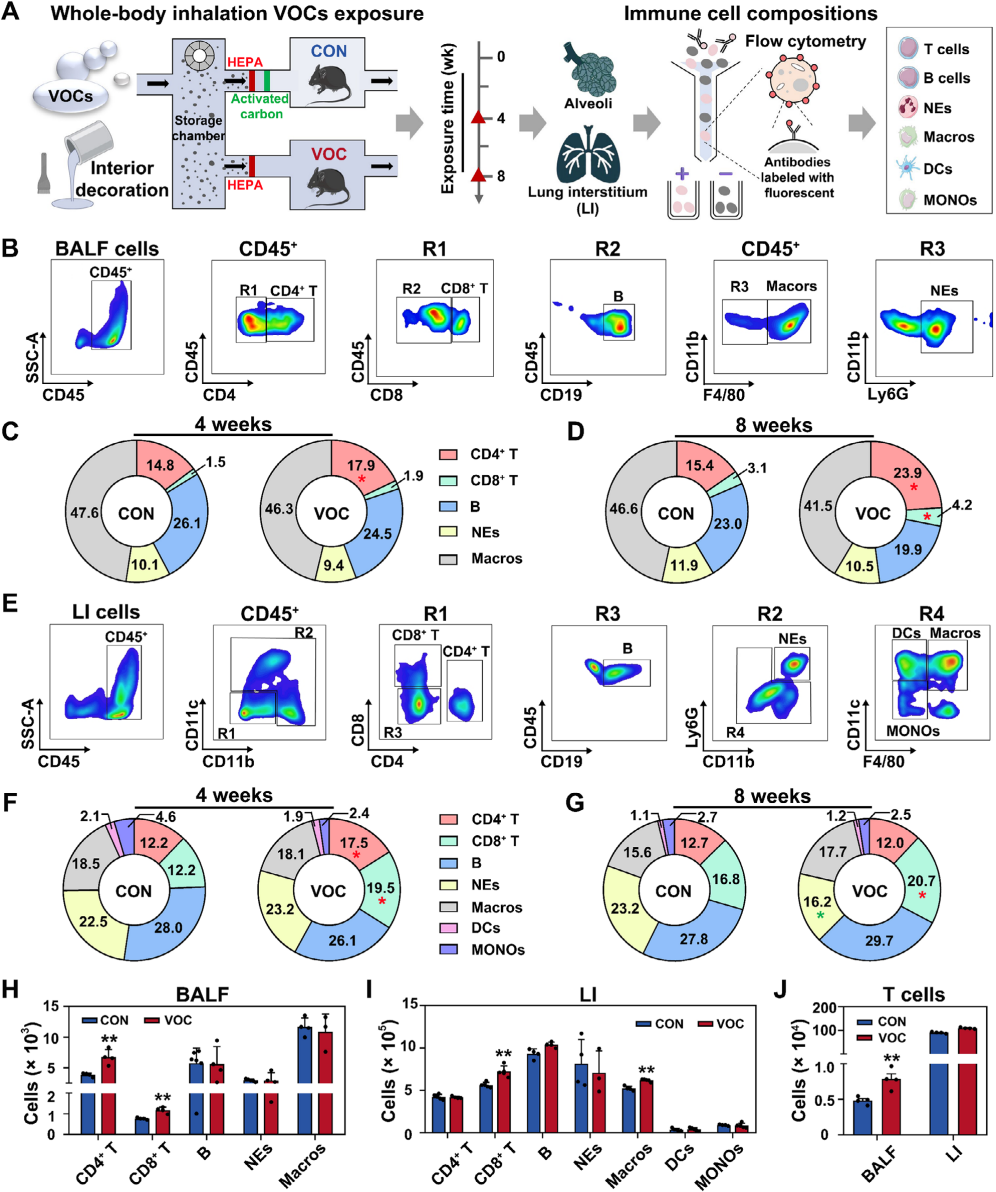
VOCs exposure disturbs immune cell composition in mice lungs. A) Experimental scheme for assessing the impact of whole‐body inhalation exposure to VOCs on the immune cell composition in the lung. B) Flow cytometric dot plots illustrating the gating strategy for the characterization of immune cell populations in the alveoli. Percentage distribution of immune cells in the alveoli after C) 4‐week or D) 8‐week exposure, comparing conditions in CON or VOC chambers. E) Representative flow cytometry plots and the corresponding quantitative analysis for percentages of immune cells derived from LI after F) 4‐week or G) 8‐week treatment. Total numbers of immune cells in the H) alveoli and I) LI upon 8‐week VOCs exposure. J) Quantitative analysis of T cells (including CD4⁺ and CD8⁺ T cells) derived from BALF and LI post‐exposure in CON or VOC chambers. Statistical analysis was performed using a two‐tailed unpaired *t*‐test (H–J). *p < 0.05 and **p < 0.01. Data are expressed as mean ± SEM (*n* ≥ 3).

C57BL/6 mice were subjected to VOCs exposure for a duration of either 4 or 8 weeks, utilizing a well‐established whole‐body inhalation exposure model designed for simulating interior decorative VOCs exposure (**Figure** **1**A). The mean VOC concentrations in the control (CON) and VOCs exposure chambers were 329.4 ± 41.0 ppbv and 717.0 ± 56.3 ppbv, respectively (Figure S1A, Supporting Information). During the exposure period, neither body weight nor organ coefficients were affected by VOCs exposure (Figure S1B–D, Supporting Information).

To investigate the effects of VOCs on immune cell composition within the lung, we performed a detailed identification of immune cell populations within both the alveoli and LI. In the alveoli, five types of immune cells, including CD4^+^ T cells, CD8^+^ T cells, B cells, NEs, and Macros were gated (Figure 1B). VOCs exposure for 4 weeks led to a proportional increase in CD4^+^ T cells relative to the control (Figure 1C). Upon 8‐week VOCs exposure, an increase was noted in the proportions of CD4^+^ T and CD8^+^ T cells (Figure 1D). In the LI, two additional types of immune cells, i.e., monocytes (MONOs) and dendritic cells (DCs), were distinguished (Figure 1E). VOCs‐exposed mice exhibited an upsurge in the proportions of CD4^+^ T cells and CD8^+^ T cells (Figure 1F), with a proportional increase in CD8^+^ T cells lasting for 8 weeks (Figure 1G).

Given that persistent changes in immune cell populations after 8‐week exposure can offer insights into disease progression,^[^ ^26^ ^]^ we then analyzed the change in immune cell counts at this exposure duration. As illustrated in Figure 1H, VOCs exposure induced a significant increase in CD4^+^ T cell counts in the alveoli, from an initial level of 3.9 × 10^3^ cells to a final level of 6.7 × 10^3^ cells, and a concomitant rise in CD8^+^ T cells from 0.8 × 10^3^ cells to 1.2 × 10^3^ cells (Table S1, Supporting Information). In the LI, CD8^+^ T cell counts were elevated from 5.6 × 10^5^ cells to 7.2 × 10^5^ cells, along with an increase in Macros (Figure 1I; Table S2, Supporting Information). Likewise, T cells, a collection containing CD4^+^ T cells and CD8^+^ T cells,^[^ ^27^ ^]^ showed an obvious increase in the alveoli and a modest rise in the LI (Figure 1J). These findings revealed that interior decorative VOCs exposure perturbed pulmonary immune cell composition, dominated by an increase in T cells, especially the elevation of CD4^+^ T cells and CD8^+^ T cells in the alveoli.

### 2.2 Enhanced T Cell Generation from Lymphoid‐Biased Hematopoiesis Induced by VOCs

**Figure 2:**
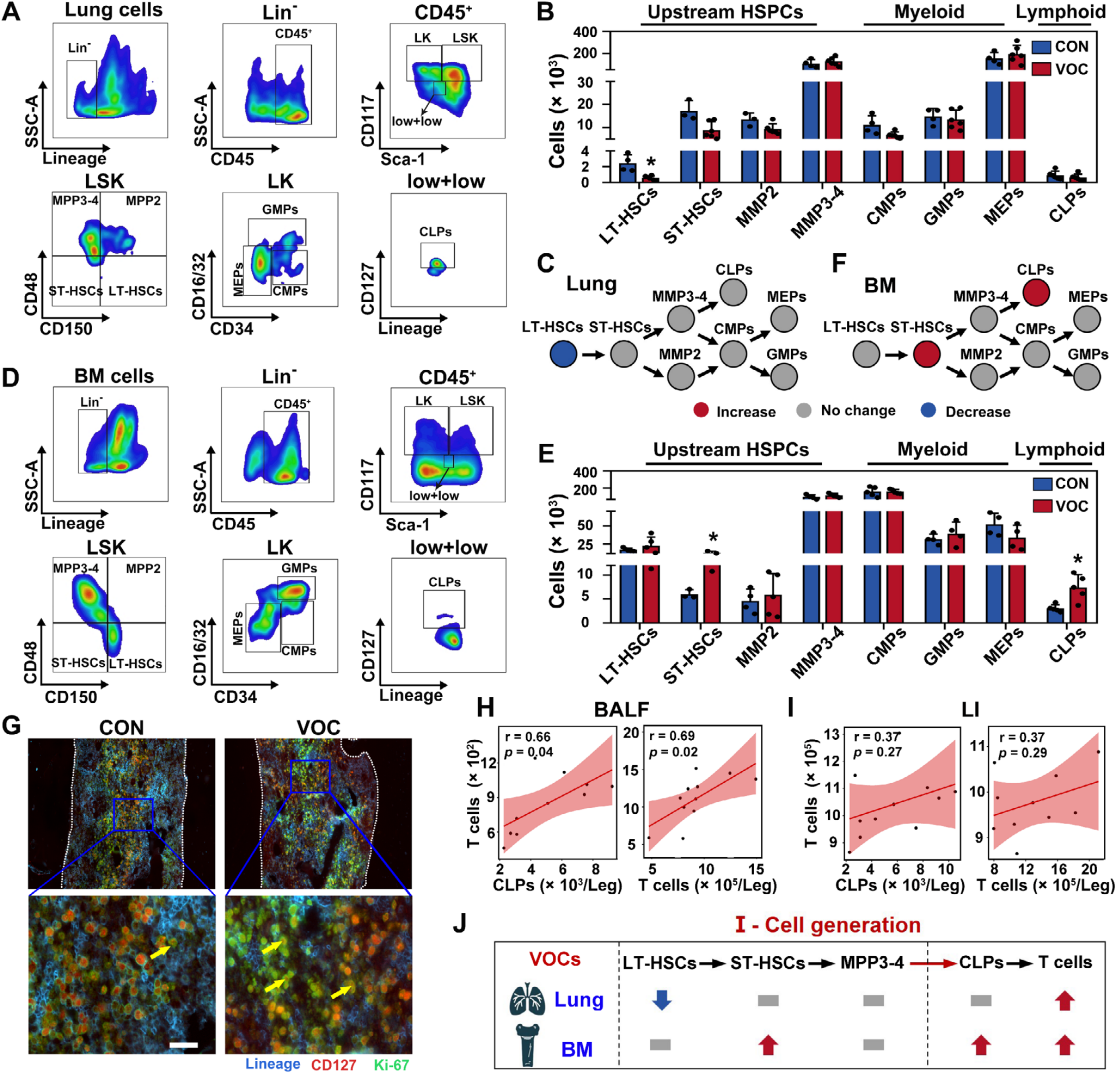
VOCs exposure promotes lymphoid‐biased hematopoiesis for T cell replenishment in the lung. A) Representative flow cytometric dot plots and B) quantitative analysis of hematopoietic cell populations derived from lung tissues of mice after 8‐week exposure in CON or VOC chambers. C) Schematic diagram illustrating the impact of 8‐week VOCs exposure on pulmonary hematopoietic process. D) Representative flow cytometric dot plots and E) quantitative analysis of hematopoietic cell populations in BM of mice after 8‐week exposure in CON or VOC chambers. F) Schematic diagram showing the effect of 8‐week VOCs exposure on BM hematopoietic process. G) Immunofluorescence analysis of proliferative CLPs stained with Lineage, CD127, and Ki‐67 specific antibodies. Scale bar: 10 µm. Correlational analysis of BM‐derived CLPs and T cells with T cells in the H) alveoli and I) LI. J) A schematic representation summarizing the alterations of key hematopoietic cells involved in T cell replenishment upon VOCs exposure. Statistical analysis was performed using two‐tailed unpaired *t*‐test (B and E) and Pearson linear correlation analysis (H and I). * *p* < 0.05. Data are expressed as mean ± SEM (*n* ≥ 3).

To investigate the underlying mechanisms underlying the increased T cell population in the lung, we initially investigated the in situ pulmonary hematopoiesis. A schematic overview of the hematopoietic process for immune cell supply is shown in Figure S2A (Supporting Information), illustrating the differentiation trajectory of hematopoietic stem cells into lineage‐committed progenitors and subsequently differentiating into LYM.^[^ ^28^ ^]^ Within this process, we identified eight distinct subsets of hematopoietic cells in the lung (**Figure** **2**A), including long‐term hematopoietic stem cells (LT‐HSCs), short‐term hematopoietic stem cells (ST‐HSCs), multipotent progenitor type 2 (MPP2), multipotent progenitor type 3–4 (MPP3‐4), common myeloid progenitors (CMPs), granulocyte‐macrophage progenitors (GMPs), megakaryocyte‐erythroid progenitors (MEPs), and common lymphoid progenitors (CLPs). Exposure to VOCs elicited a significant activation of pulmonary hematopoiesis at 4‐week intervals (Figure S2B,C and Table S3, Supporting Information). This stood in sharp contrast to the 8‐week exposure, where we observed an inhibition of hematopoiesis, as evidenced by the abnormal reduction in LT‐HSCs (Figure 2B,C; Table S4, Supporting Information). These results suggest a limited role for pulmonary hematopoiesis in the augmented T cell population in the lung after 8‐week VOCs exposure.

Our investigation thus shifted focus to examine hematopoiesis within the distal BM. Similar to pulmonary hematopoiesis, we identified various hematopoietic cells in the BM (Figure 2D). Compared with the CON group, VOCs‐exposed mice displayed the elevations of LT‐HSCs, ST‐HSCs, MPP3‐4, and CLPs numbers at 4 weeks (Figure S2D and Table S5, Supporting Information), suggesting the initiation and activation of the hematopoietic process (Figure S2E, Supporting Information). Of note, mice continuously exposed to VOCs for 8 weeks still induced a 2.1‐fold increase in ST‐HSCs and a 2.4‐fold increase in CLP numbers (Figure 2E; Table S6, Supporting Information), indicating a sustained and selective bias toward lymphoid hematopoiesis in the BM (Figure 2F). Moreover, CLPs, which are lineage‐committed progenitors serving as a reservoir for LYM,^[^ ^29^ ^]^ exhibited an elevation in the proliferative capacity (Figure 2G), confirming the occurrence of lymphoid‐biased hematopoiesis in the BM.

To determine whether the hematopoiesis in the BM was responsible for the continuous elevation of T cell population in the lung, we initially assessed the generation of T cells derived from BM lymphoid‐biased hematopoiesis. Our observation revealed a significant increase in T cell numbers in the BM (Figure S2F, Supporting Information). Subsequently, we performed a correlational analysis to probe the associations between BM‐derived T cell supplement, including CLPs and T cells in the BM, and T cell counts in the lung. As depicted in Figure 2H, a robust positive correlation was identified between the T cell counts in the alveoli and the numbers of CLPs (r = 0.66, *p* = 0.04) or T cells (r = 0.69, *p* = 0.02) in the BM. This correlation indicated a supply of T cells from lymphoid‐biased hematopoiesis within the BM to the alveoli. Nevertheless, a slight correlation was observed between the T cell counts in the LI and the BM‐resident cells (Figure 2I), suggesting a less significant supply of T cells to this region. Accordingly, interior decorative VOCs exposure for 8 weeks promoted lymphoid‐biased hematopoiesis in the BM, resulting in T cell generation and their subsequent supplement to target lung tissues, which were preferentially supplied to the alveoli (Figure 2J).

### 2.3 T Cell Maturation and Recruitment to the Lung Following VOCs Exposure

**Figure 3:**
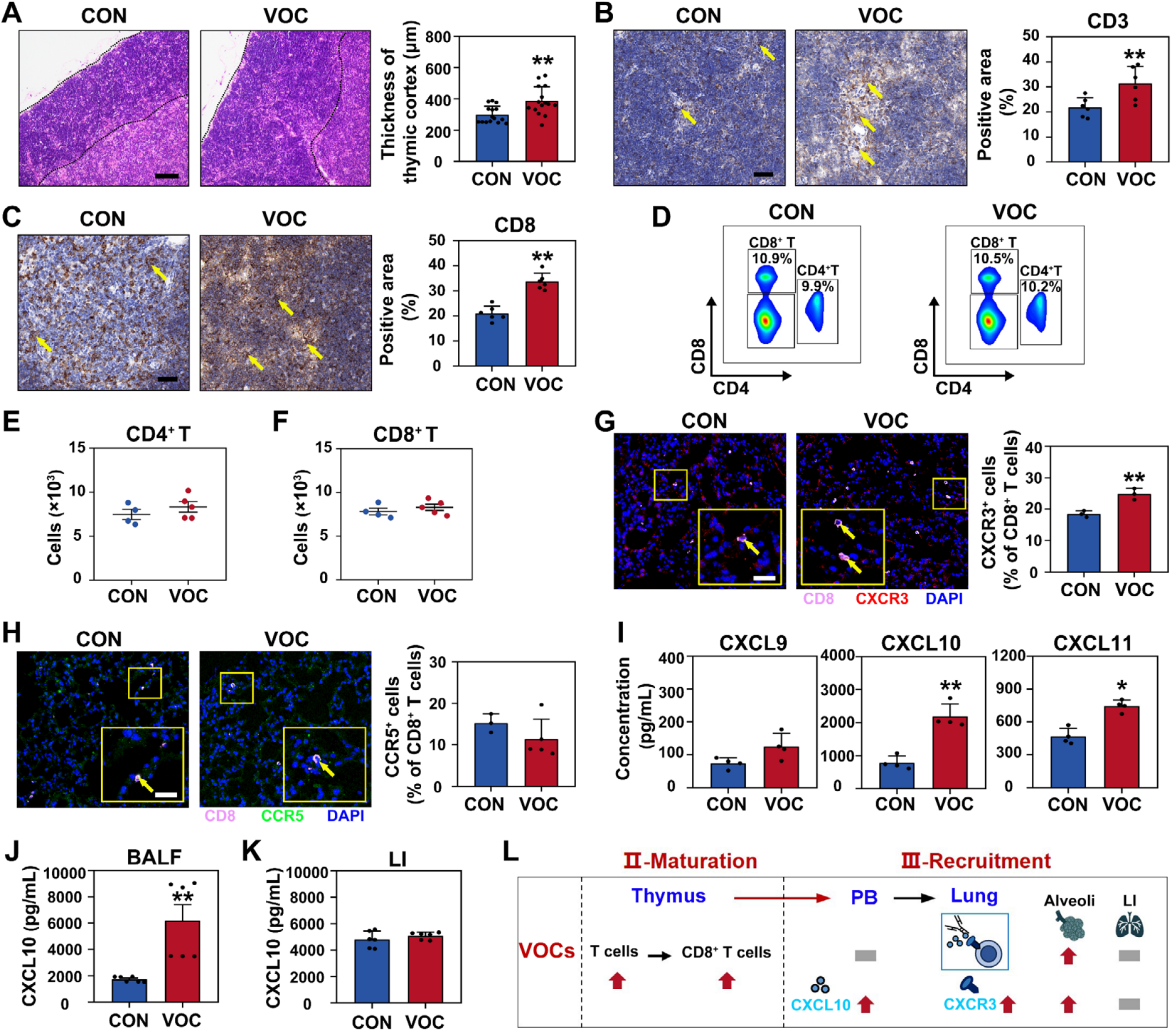
VOCs exposure enhances T cell maturation and their recruitment to lung tissues. Mice were exposed to CON or VOCs for 8‐week using the whole‐body inhalation exposure model. A) Representative images of H&E‐stained thymic sections. Black dashed lines indicate the cortical thickness of thymus. Scale bar: 100 µm. Immunohistochemical analysis for B) CD3 and C) CD8 in thymic sections, and the percentage of positive area. Yellow arrows indicate the positive expression. Scale bar: 40 µm. D) Representative flow cytometric dot plots and quantitative analysis of E) CD4^+^ T and F) CD8^+^ T cells in the PB. G) Immunostaining for the colocalization of CXCR3 (red) and CD8 (purple) in lung sections, and the corresponding quantitative analysis. Yellow arrows indicate CXCR3 colocalization with CD8. Scale bar: 20 µm. H) Immunostaining for the colocalization of CCR5 (green) and CD8 (purple) in lung sections, and the corresponding quantitative analysis. Yellow arrows indicate CCR5 colocalization with CD8. Scale bar: 20 µm. I) Quantitative analysis of CXCL9, CXCL10, and CXCL11 in the PB by ELISA assay. Protein expressions of CXCL10 in J) BALF and K) LI. L) A schematic representation summarizing the T cell maturation and recruitment to lung tissue. Statistical analysis was performed using two‐tailed unpaired *t*‐test (A–C and E–K). * *p* < 0.05 and ** *p* < 0.01. Data are expressed as mean ± SEM (*n* ≥ 3).

Considering that BM‐derived T cells undergo maturation before entering into lung tissues (Figure S3, Supporting Information), we then determined the impact of VOCs exposure on T cell maturation by measuring the thickness of the thymic cortex, a proxy for T cell maturation.^[^ ^30^ ^]^ As shown by **Figure** **3**A, exposure to VOCs was associated with a significant thickening of the thymic cortex, suggesting a potential facilitation of T cell maturation by VOCs. To corroborate the enhancement in T cell maturation, we evaluated the expression levels of mature T cell markers (i.e., CD3 and CD8) in the thymus. VOCs‐exposed mice exhibited a substantial elevation in both CD3 (≈1.4‐fold; Figure 3B) and CD8 (≈1.6‐fold; Figure 3C) expression relative to the CON group, indicating a promoted maturation of T cells in the thymus.

To assess the recruitment of mature T cells to the lung, we first examined T cell counts in the peripheral blood (PB), a necessary step for the recruitment of cells from generation sites to target tissues. Circulating T cell numbers, including CD4^+^ T and CD8^+^ T cells, remained unchanged following VOCs exposure (Figure 3D–F; Table S7, Supporting Information), suggesting that mature T cells might be recruited from the PB to lung tissue. To test this hypothesis, we investigated the expression levels of C–X–C motif chemokine receptor 3 (CXCR3) and C–C motif chemokine receptor 5 (CCR5) in lung tissues, which are crucial receptors for T cell recruitment.^[^ ^31^ ^]^ As indicated by yellow arrows in Figure 3G, VOCs‐exposed mice showed an increased expression of CXCR3 (red) around mature T cells stained with CD8 (purple), suggesting that CXCR3 emerged as a pivotal receptor mediating the recruitment of mature T cells to the lung in response to VOCs exposure. In contrast, VOCs‐exposed mouse lung did not display a significant alteration in CCR5 (green) level as compared to the control (Figure 3H). Next, we measured the serum levels of key CXCR3 ligands, including C–X–C motif chemokine ligand 9 (CXCL9), CXCL10, and CXCL11. VOCs exposure induced a 2.8‐fold increase in CXCL10 and a 1.6‐fold increase in CXCL11 levels, with CXCL10 showing the most pronounced upregulation (Figure 3I). Consistently, VOCs‐exposed mice exhibited a 3.6‐fold increase in the level of CXCL10 in the alveoli (Figure 3J) but not LI (Figure 3K), indicating enhanced recruitment of T cells to the alveolar compartment. Together, interior decorative VOCs exposure promoted T cell maturation in the thymus and facilitated their recruitment from circulation into lung tissues, especially toward the alveoli (Figure 3L).

### 2.4 Role of Osteogenic Differentiation for BM Lymphoid‐Biased Hematopoiesis

**Figure 4:**
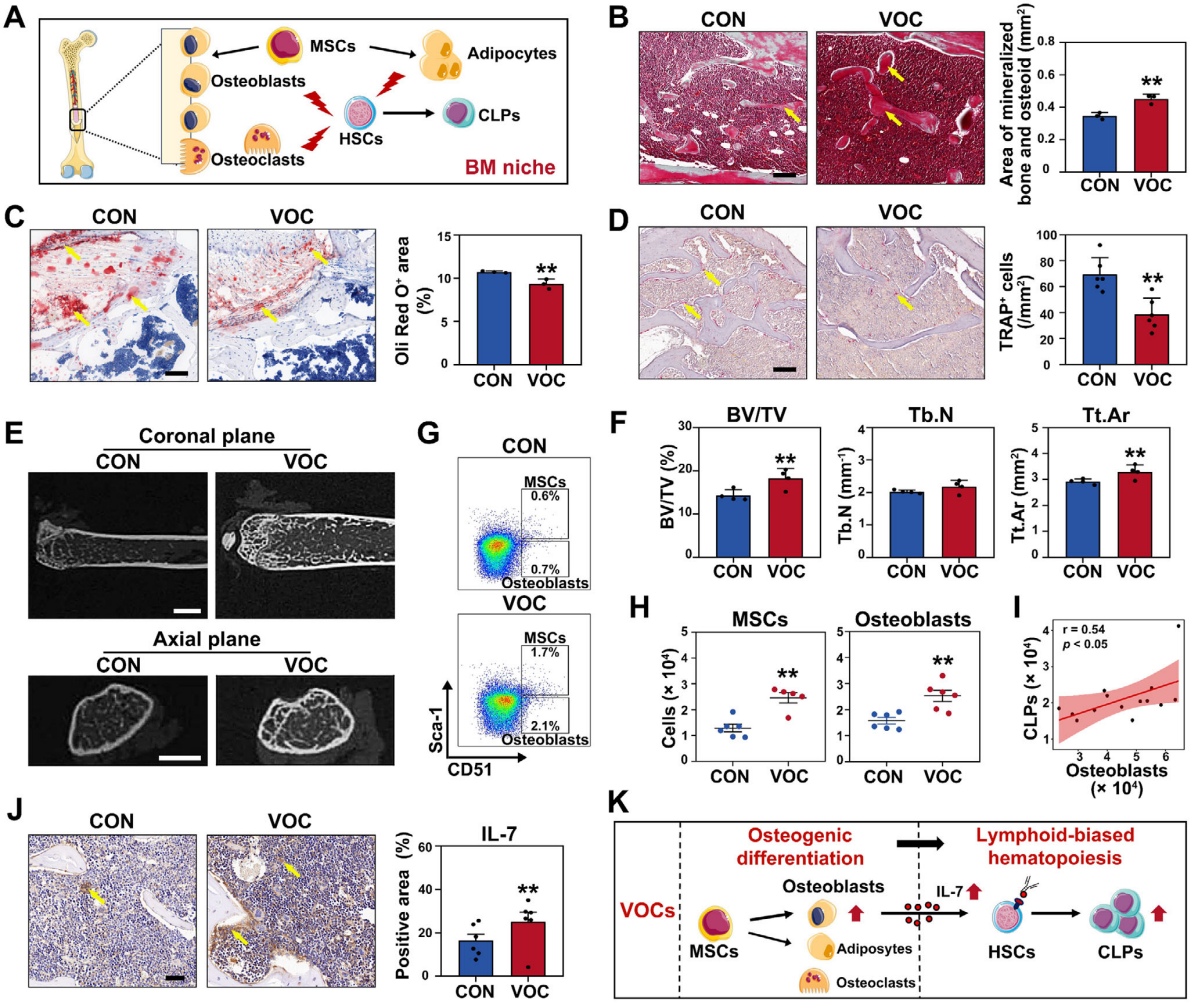
VOCs exposure promotes lymphoid‐biased hematopoiesis stimulated by osteogenic differentiation in the BM niche. Mice were exposed to CON or VOCs for an 8‐week duration utilizing a whole‐body inhalation exposure model. A) Crucial cellular components in BM niche involved in promoting lymphoid‐biased hematopoiesis. Representative images and quantifications of femur sections stained with B) Golder trichrome, C) Oil red O, and D) TRAP staining. Yellow arrows indicate osteoid formation, osteoclast formation, and adipogenic formation, respectively. Scale bar: 100 µm. E) Representative images of micro‐CT and F) quantitative analysis of Tb. N, BV/TV, and Tb. Sp for mouse femur. Scale bar: 1 mm (up) and 500 µm (down). G) Representative flow cytometric dot plots and H) quantitative analysis of MSCs and osteoblasts in BM niche of mice. I) Correlational analysis of osteoblasts in BM niche with BM‐derived CLPs. J) Representative images of immunohistochemical staining for IL‐7 and corresponding quantification of positive area. Yellow arrows indicate positive expression positive area. Scale bar: 40 µm. K) A schematic representation summarizes the promotion of osteogenic differentiation of BM in lymphoid‐biased hematopoiesis upon VOCs exposure. Statistical analysis was performed using two‐tailed unpaired t‐test (B–D, F, H, and J) and Pearson linear correlation analysis (I). * *p* < 0.05 and ** *p* < 0.01. Data are expressed as mean ± SEM (*n* ≥ 3).

To elucidate the regulatory mechanisms driving T cell replenishment, we then investigated the initial phase, i.e., BM hematopoiesis biased toward lymphoid lineage, which indirectly influenced the subsequent phases of T cell renewal. Given that crucial cellular components (i.e., osteoblasts, adipocytes, and osteoclasts) in BM niche play an attributable role in the lymphoid‐biased hematopoiesis,^[^ ^32^ ^]^ their formation within this niche was examined (**Figure** **4**A). As depicted by yellow arrows in Figure 4B, Golder trichrome‐stained femurs from VOCs‐exposed mice exhibited an increase in the formation of uncalcified newly‐synthesized bone matrix (Figure 4B), suggesting the active osteoblastic bone formation in BM niche. In contrast, a decrease in Oil Red O‐positive area was observed in VOCs‐exposed mice (Figure 4C), indicating the reduction of adipogenic precursors or mature adipocytes. Moreover, VOCs‐exposed mice displayed a decline in osteoclast formation within BM niche (indicated by TRAP‐positive area in Figure 4D). These findings across the above three cellular components suggest the stimulation of osteogenic differentiation in the BM niche by VOCs exposure, which may play a crucial role in VOCs‐induced lymphoid‐biased hematopoiesis in the BM.

To further substantiate the osteogenic differentiation stimulated by VOCs, we performed a micro‐computed tomography (Micro‐CT) assessment of the mouse femur to detect the osteoblastic bone formation in the BM niche (Figure 4E). As anticipated, key parameters indicative of skeletal architecture, such as bone volume fraction (BV/TV) and total bone area (Tt.Ar), were significantly increased in VOCs‐exposed mice when compared to the mice in CON group (Figure 4F). Quantification of upstream mesenchymal stem cells (MSCs) and osteoblasts showed 1.9‐ and 1.6‐fold increase following VOCs exposure (Figure 4G, H, Figure S4A, and Table S8, Supporting Information), with a corresponding enhancement in the mRNA level of the osteogenic differentiation gene *Runx2* (Figure S4B, Supporting Information), confirming the promotion of osteogenic differentiation promoted by VOCs.

We next investigated the correlation between osteoblasts and lineage‐committed progenitors (i.e., CLPs) in the BM. As depicted in Figure 4I, the number of CLPs was highly associated with osteoblasts in BM niche, indicating a potential role of osteoblasts in the promotion of BM hematopoiesis toward lymphoid lineage. To further examine the role of osteoblasts in this process, we detected the levels of IL‐7 and CXCL12, two well‐recognized osteoblast‐derived cytokines involved in hematopoiesis,^[^ ^33^, ^34^ ^]^ in the BM niche. As indicated by the yellow arrows in Figure 4J, the femur tissues from VOCs‐exposed mice exhibited an increase in the protein level of IL‐7, confirming the essential role of osteoblasts in facilitating lymphoid‐biased hematopoiesis in the BM upon VOCs exposure. In contrast, no statistical alteration was observed in CXCL12 level, implying CXCL12 was not the specific cytokine that responded to VOCs exposure (Figure S5, Supporting Information). These data indicated that interior decorative VOCs exposure stimulated osteogenic differentiation in the BM niche, thereby promoting the lymphoid‐biased hematopoietic fate (Figure 4K).

### 2.5 Regulatory Role of IL‐6 and IL‐17A in Osteogenic Differentiation in BM Niche

**Figure 5:**
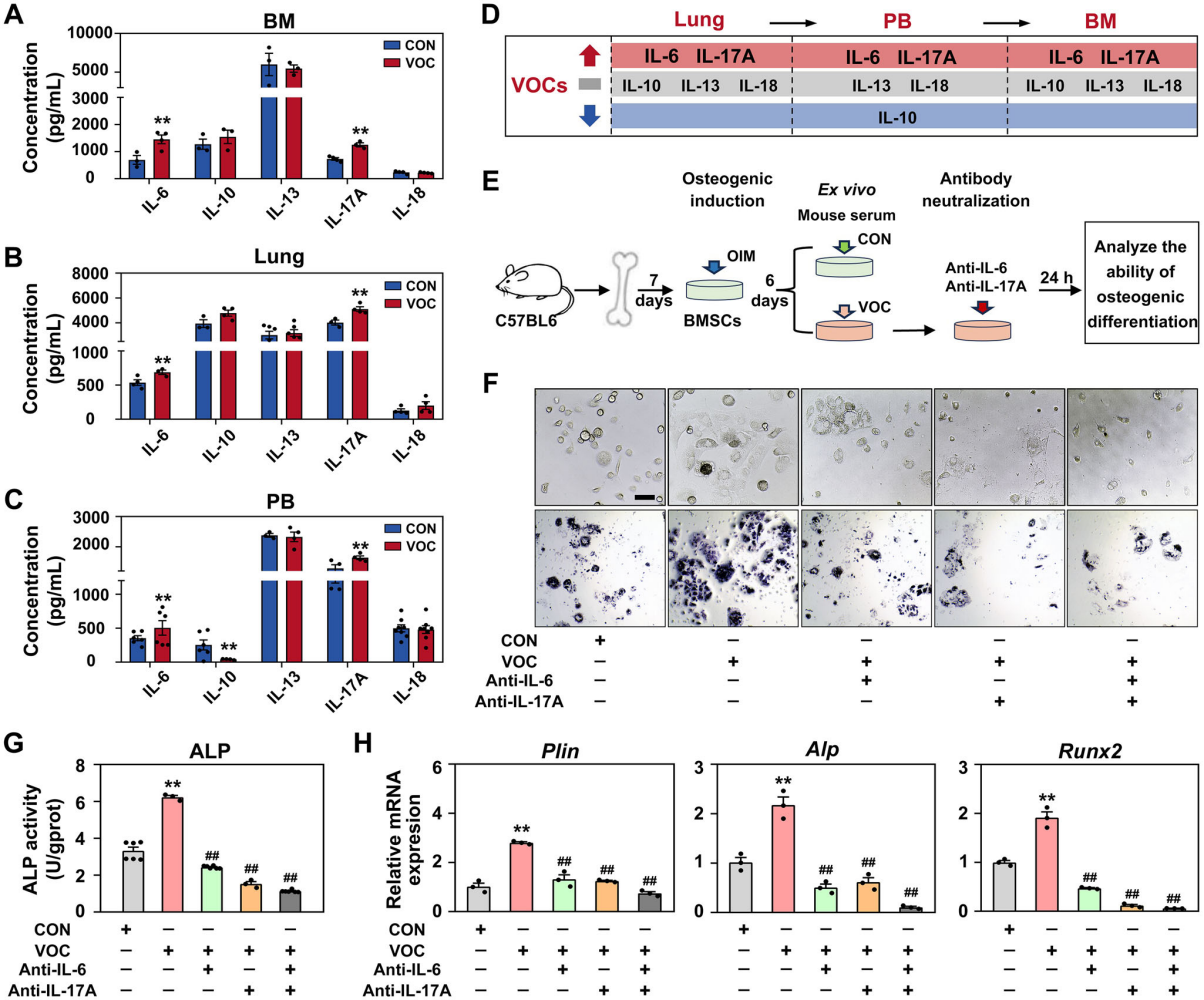
VOCs exposure induces BMSCs into an osteogenic fate via the mediation of IL‐6 and IL‐17A. Quantitative analysis of IL‐6, IL‐10, IL‐13, IL‐17A, and IL‐18 in the A) BM, B) lung tissues, and C) PB by ELISA. D) A schematic representation summarizes the alterations of the above cytokines in three sites upon VOCs exposure. E) Design for the osteogenic induction, ex vivo biosensor assay, and antibody neutralization tests. Osteogenesis was determined by F) ALP staining and G) ALP activity assays. Scale bar: 400 µm. H) Relative mRNA levels of *Plin*, *Alp*, and *Runx2* in mouse BMSCs. Statistical analysis was performed using two‐tailed unpaired t‐test (A–C) and one‐way ANOVA (G,H). * *p* < 0.05 and ** *p* < 0.01 versus the CON group. ***^#^*** *p* < 0.05 and ***^##^*** *p* < 0.01 versus VOCs‐exposed group. Data are expressed as mean ± SEM (*n* ≥ 3).

To uncover the mechanisms governing osteogenic differentiation in the BM niche of mice exposed to VOCs, we then focused on key cytokines implicated in this process.^[^ ^35^, ^36^ ^]^ Among the interleukins examined—IL‐6, IL‐10, IL‐13, IL‐17A, and IL‐18—only IL‐6 and IL‐17A levels were significantly elevated in the BM of VOCs‐exposed mice compared to controls (**Figure** **5**A), suggesting their critical role in niche osteogenesis. Given that interleukins are predominantly produced in inflamed areas,^[^ ^37^ ^]^ the lung, as the primary site of cytokine synthesis upon inhalational exposure, is likely a major contributor to these cytokines. This was supported by the selective increase in IL‐6 and IL‐17A, but not other cytokines, in the lung following VOCs exposure (Figure 5B). Moreover, elevated levels of IL‐6 and IL‐17A were detected in the PB (Figure 5C). These data suggest that IL‐6 and IL‐17A are central to enhancing osteogenic differentiation within the BM niche, with their levels elevated across the BM, lung, and PB (Figure 5D).

To identify the key cytokines mediating VOCs‐induced osteogenic differentiation, we initiated an ex vivo biosensor assay using circulating serum (Figure 5E), a readily accessible sample that traces cytokine migration from source to target tissues. Bone marrow mesenchymal stem cells (BMSCs) treated with serum from VOCs‐exposed mice under osteogenic induction showed enhanced alkaline phosphatase (ALP) levels and activity, signifying osteogenesis, compared to controls (Figures 5F, G and S6, Supporting Information), indicating potentiated osteogenic differentiation by VOCs. Consistently, the upregulated mRNA expressions of osteogenic differentiation genes, including *Plin*, *Alp*, and *Runx2*, confirmed the potentiation of osteogenic differentiation by VOCs‐exposed serum (Figure 5H). Next, we tested the direct effects of IL‐6 and IL‐17A on VOCs‐induced osteogenic differentiation using neutralizing antibodies against each cytokine alone or both (Figure 5E). The addition of anti‐IL‐6 and anti‐IL‐17A antibodies to VOCs‐exposed serum attenuated ALP levels and activity compared to serum treatment alone, with the greatest suppression observed upon their combined application (Figure 5F,G). Moreover, BMSCs treated with anti‐IL‐6 and anti‐IL‐17A in the presence of VOCs‐exposed serum exhibited a marked reduction in the mRNA expressions of *Plin*, *Alp*, and *Runx2*, with the combined antibody treatment showing a more pronounced reduction (Figure 5H). This demonstrated that IL‐6 and IL‐17A had a direct and synergistic role in determining the osteogenic fate of BMSCs following VOCs exposure.

### 2.6 Increased Risks of VOCs‐Induced Obstructive Lung Diseases via the Mediation of Lymphocytes

**Figure 6:**
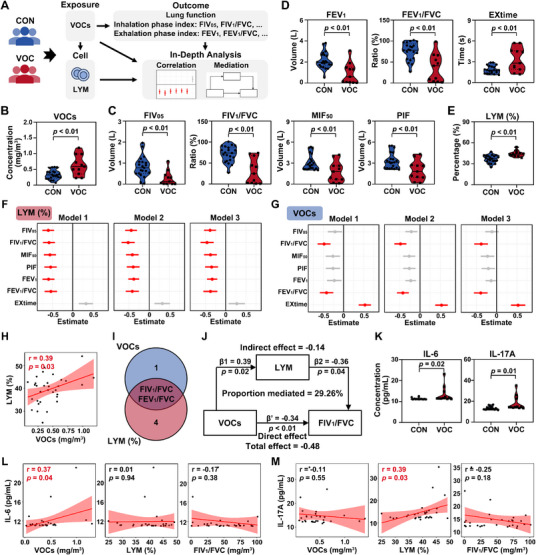
VOCs exposure elevates the risks of obstructive lung diseases via the mediation of LYM in the population cohort. A) Schematic representation of population cohort study. B) Concentrations of VOCs in CON and VOCs‐exposed populations. C) Inhalation phase index of lung function, including FIV_05_, FIV_1_/FVC, MIF_50_, and PIF, was measured by a pulmonary function testing machine. D) Results of exhalation phase index (FEV_1_, FEV_1_/FVC, and EXtime) in humans. E) Quantitative assessment of the proportion of LYM in the PB. Forest plots indicating the correlation between lung function parameters and F) LYM proportions or G) VOC concentrations, respectively. Model 1: Without adjustment. Model 2: Adjusted for age. Model 3: Model 2 with additional adjustment for BMI, smoking status, and alcohol drinking status. H) Linear correlation analysis of VOC concentrations and LYM proportions. I) The overlap of lung function indicators that displayed significant correlations with VOC concentrations and LYM proportion. J) Path diagram of mediation analysis of LYM proportion in the overall reduction in FIV_1_/FVC induced by VOCs. K) Quantitative analysis of IL‐6 and IL‐17A in human plasma by ELISA. Linear correlation analysis of L) IL‐6 and M) IL‐17A with VOC concentrations, LYM proportions, and FIV_1_/FVC. Data were presented as mean ± SD or median (IQR), with sample sizes of *n* = 21 for the CON group and *n* = 11 for the exposure group. Statistical analysis was performed using two‐tailed unpaired *t*‐test (B–D and K), linear regression (F, G, and J), and Pearson linear correlation analysis (H, L, and M).

To define the detrimental respiratory impacts of inhalational VOCs, we carried out a population‐based cohort study (**Figure** **6**A; Table S9, Supporting Information). Compared with the control, VOCs‐exposed populations resided in environments with higher VOC concentrations (Figure 6B). These individuals showed a reduction in forced inspiratory volume in 0.5 s (FIV_05_), the ratio of forced inspiratory volume in 1 s to forced vital capacity (FIV_1_/FVC), maximal inspiratory flow at 50% (MIF_50_), and peak inspiratory flow (PIF), the inhalation phase index for assessing lung ventilation function (Figure 6C; Table S10, Supporting Information), indicating the occurrence of ventilatory dysfunction. Forced expiratory volume in 1 s (FEV_1_) and FEV_1_/FVC, two exhalation phase indicators to assess ventilatory dysfunction, were decreased following VOCs exposure (Figure 6D; Table S10, Supporting Information). Meanwhile, an increase was observed in exhalation time (EXtime) (Figure 6D; Table S10, Supporting Information). Moreover, VOCs led to a slight decrease in several indicators of expiratory flow (Figure S7 and Table S10, Supporting Information). Consistent with these human findings, VOCs exposure led to functional impairment and structural damage of the mouse lung (Figure S8, Supporting Information). Therefore, exposure to VOCs resulted in obstructive ventilation dysfunction, thereby increasing the risks of obstructive lung diseases.

To further determine whether VOCs‐increased T cells contribute to lung adverse outcomes, we thus detected LYM, a collection comprising all types of T cell subsets,^[^ ^27^ ^]^ within human PB. In comparison with the CON group, both the proportion and number of LYM were elevated in the PB from VOCs‐exposed populations (Figure 6E; Figure S9A, Supporting Information). Subsequently, we constructed three adjusted regression models to investigate the correlation between LYM and lung function metrics. As illustrated in Figure 6F, an increase in LYM proportions was highly associated with a decrease in FIV_05_, FIV_1_/FVC, MIF_50_, PIF, FEV_1_, and FEV_1_/FVC (all *p* < 0.05, and Table S11, Supporting Information). In contrast, no significant association was observed between LYM numbers and lung function parameters, except for FIV_1_/FVC (Figure S9B and Table S12, Supporting Information). Moreover, VOC concentrations were negatively associated with FIV_1_/FVC and FEV_1_/FVC, but positively associated with EXtime (all *p* < 0.05, Figure 6G; Table S13, Supporting Information). The concentrations of VOCs were also positively associated with LYM proportions (r = 0.39, *p* = 0.03) (Figure 6H). As depicted in Figure 6I, both LYM proportion and VOC concentrations were highly associated with two crucial indicators, i.e., FIV_1_/FVC and FEV_1_/FVC, hinting at the mediating role of LYM in VOCs‐induced lung injury.

A mediation analysis was then conducted to explore the role of LYM in mediating the decline in FIV_1_/FVC and FEV_1_/FVC induced by VOCs. The total effect of VOCs on FIV_1_/FVC was −0.48 (β (95% CI): −0.48 (−0.90, −0.25), *p* < 0.01), with the direct effect of VOCs being −0.34 (β (95% CI): −0.34 (−0.79, −0.10), *p* < 0.01) and the indirect effect mediated by LYM being −0.14 (β (95% CI): −0.14 (−0.30, −0.02), *p* = 0.01) (Figure 6J; Table S14, Supporting Information). A notable indirect effect was detected for LYM, contributing to 29.26% of the overall reduction in FIV_1_/FVC through the mediating influence of LYM (Figure 6J and Table S14, Supporting Information). Meanwhile, the mediating effect of LYM contributed to 25.05% of the overall reduction in FEV_1_/FVC, which did not attain statistical significance (Table S15, Supporting Information).

In addition to the evaluation of lung function and LYM, IL‐6 and IL‐17A levels were also measured in the plasma samples. As shown in Figure 6K, VOCs‐exposed populations exhibited higher levels of IL‐6 and IL‐17A relative to the control. We then determined their correlation with VOC concentrations, LYM proportions, and FIV_1_/FVC. The level of IL‐6 was positively associated with VOC concentrations (r = 0.35, *p* = 0.04), while exhibiting no significant association with LYM proportions and FIV_1_/FVC (Figure 6L). Moreover, an increase in IL‐17A levels was only associated with elevated LYM proportions (r = 0.36, *p* = 0.03) (Figure 6M). These results hinted at their potential role as targets for mitigating VOCs‐related obstructive lung diseases. Accordingly, interior decorative VOCs‐associated obstructive lung diseases were potentially mediated by the elevation of LYM and regulated by IL‐6 and IL‐17A.

## 3 Discussion

The escalating prevalence of interior decoration has elevated indoor VOC levels and attendant lung disease risks.^[^ ^4^ ^]^ However, existing studies have largely focused on individual or dominant VOC species rather than real‐world mixtures, and the mechanisms underlying VOCs‐associated lung risks remain elusive. To address this gap, we establish a whole‐body inhalation exposure mouse model and reveal that interior decorative VOCs exposure for 4 weeks increases alveolar T cells (CD4^+^ T and CD8^+^ T cells), which persists until 8 weeks. Notably, while 4‐week exposure enhances hematopoiesis in both local lung and distal BM, 8‐week exposure drives selective BM hematopoiesis with pronounced lymphoid lineage commitment. This temporal shift establishes BM lymphoid‐biased hematopoiesis rather than local lung as the principal contributor to alveolar T cell persistence. VOCs‐induced osteogenic differentiation within the BM niche, driven by IL‐6 and IL‐17A, promotes BM hematopoiesis toward the lymphoid axis. Our population‐based cohort study links VOCs‐expanded LYM to elevated obstructive lung disease risk, with exposed individuals showing higher IL‐6 and IL‐17A levels (Figure S10, Supporting Information).

Our whole‐body inhalation exposure model effectively recapitulates real‐world VOC exposure scenarios, overcoming the limitations of prior studies focused on individual or dominant VOC species. Among the identified 116 VOC species,^[^ ^10^ ^]^ the top 10 high‐abundance components are listed in Table S16 (Supporting Information). Comparative analysis revealed a distinct compositional profile in the VOCs‐exposed group, comprising 39.67% aromatic hydrocarbons, 31.48% halogenated hydrocarbons, 22.23% alkanes, and 6.62% oxidized VOCs.^[^ ^38^ ^]^ This profile aligns with a prior study on newly renovated homes, which also identified these VOC species as the predominant contributors to adverse health outcomes.^[^ ^12^ ^]^ Crucially, our study demonstrates a significant association between VOCs exposure and obstructive lung disease risk. This finding is corroborated by existing epidemiological evidence. For instance, a cohort study of 1,477 subjects reported a positive association between VOC concentrations and chronic obstructive pulmonary disease (COPD) risk.^[^ ^39^ ^]^ Furthermore, recent analyses have linked specific blood VOCs (e.g., 1,4‐dichlorobenzene, m‐/p‐xylene, bromodichloromethane, and nitromethane) to pulmonary impairment.^[^ ^40^ ^]^

As the initial sentinels against inhaled insults,^[^ ^41^ ^]^ pulmonary immune response is a major driver in lung disease pathogenesis,^[^ ^42^ ^]^ with spatial immune regulation shaping disease‐specific microenvironments. Our systematic investigation of the lung microenvironment reveals that VOCs exposure induces pulmonary immune dysregulation dominated by T‐cell predominance, with consecutive increases in alveolar CD4^+^ and CD8^+^ T cells. Given the clinical impracticality of invasive lung‐specific T‐cell measurements in humans, we employed circulating LYM as surrogate markers. To ensure analytical consistency across all participants, we utilized standardized complete blood cell counts, which offer both cost efficiency and practical advantages by avoiding complex sample handling and potential inter‐batch variability. It should be acknowledged, however, that this approach does not allow for precise immune cell classification. Importantly, our human data align with animal findings, demonstrating that circulating LYM mediate VOCs‐induced obstructive ventilation dysfunction, a hallmark of COPD. Although our cohort size may limit epidemiological generalizability, the consistency between human trends and experimental results provides compelling evidence for the biological relevance of VOC‐induced immunotoxicity in respiratory impairment. Of note, CD8^+^ T cells can act as a bridge between inhalation exposure and airway obstruction, with greater contributions than CD4^+^ T cells.^[^ ^43^ ^]^ As the predominant T‐cell population in both airways and alveolar compartments of COPD patients,^[^ ^44^ ^]^ CD8^+^ T cells drive airway remodeling and represent promising biomarkers for assessing airflow limitation and emphysema progression.^[^ ^45^ ^]^

Our study establishes a novel spatiotemporal paradigm of pulmonary immune replenishment by delineating the contributions of local lung niches and distal BM reservoirs. During 8 weeks of VOC exposure, we observed a distinct transition from pulmonary to BM‐dominated hematopoiesis. This shift may reflect the greater stability of BM hematopoietic cells than their lung counterpart, which are susceptible to functional exhaustion under chronic stress via pathways like Wnt/β‐catenin.^[^ ^46^, ^47^ ^]^ A recent study reported that human lung‐derived HSCs engrafted in mouse BM upon transplantation,^[^ ^48^ ^]^ indicating a potential for trafficking between these compartments. Although prior studies have documented the capacity of major VOC components to stimulate BM hematopoiesis,^[^ ^21^, ^22^ ^]^ our research provides insight into the subsequent trafficking of these BM‐derived cells to target lung tissues. Lymphoid‐biased hematopoiesis in the BM served as a continuous source of immature T cells into the thymus, a central site in mediating T cell maturation and selection.^[^ ^49^ ^]^ Then, mature T cells are recruited into target tissues in response to various stimuli, a process mediated by chemokines and their receptors.^[^ ^50^ ^]^ CXCL10, acting as a potent chemoattractant, binds to its receptor CXCR3 expressed on activated T cells.^[^ ^51^ ^]^ This interaction directs the migration of CXCR3^+^ T cells along a chemotactic gradient from circulation toward target sites.^[^ ^52^ ^]^ Our experimental findings, supported by existing literature evidence, reveal that VOCs‐enhanced lymphoid hematopoiesis in the BM contributes to pulmonary T cell accumulation. However, state‐of‐the‐art methods—such as suppressing BM hematopoiesis and lineage tracing in chimeric mouse models^[^ ^53^, ^54^ ^]^—will be essential to definitively confirm this proposed mechanistic pathway.

Hematopoietic regulation involves complex molecular mechanisms governed by intricate pathways, feedback loops, and cytokine networks.^[^ ^55^ ^]^ Among these, the BM niche has emerged as a critical determinant in lymphoid fate decisions.^[^ ^32^ ^]^ In this context, our data demonstrate that VOCs exposure promotes osteogenic differentiation within the BM niche—a change established to support lymphoid hematopoiesis^[^ ^56^ ^]^—revealing a niche‐focused pathway underlying VOCs‐induced lymphoid bias. Within the complex cytokine networks, we reveal that IL‐6 and IL‐17A are key mediators of this specific osteogenic process, with a synergistic contribution to creating a lymphoid‐supportive niche. This finding aligns with previous work that in vitro replenishment of IL‐6 and IL‐17A enhanced osteogenic differentiation by improving the OPG/RANKL ratio and MC3T3‐E1 cell adhesion on hydroxyapatite.^[^ ^36^ ^]^ Notably, high‐resolution techniques such as single‐cell RNA‐seq will help further characterize the changes in HSCs and BM niche, elucidating cellular and molecular interactions underlying these processes. While our study focuses on the contribution of IL‐6 and IL‐17A to BM niche remodeling, their associations in our human cohort may also reflect the broader and established roles in exerting context‐dependent effects. The literature‐based evidence posits that IL‐6 acts as an early sensor of injury,^[^ ^57^ ^]^ while IL‐17A is directly involved in immune cell recruitment and inflammatory lung pathology.^[^ ^58^, ^59^ ^]^ Moreover, our findings provide preliminary support for the application of existing inhibitors targeting IL‐6 and IL‐17A for VOCs‐induced lung disease. This is consistent with clinical evidence showing that IL‐6 blockade improved airflow limitation in COPD patients^[^ ^60^ ^]^ and preclinical data demonstrating that IL‐17A knockdown mitigated inflammatory lung injury.^[^ ^61^ ^]^

Based on our experimental data and literature‐based evidence, we propose a mechanistic pathway wherein VOCs exposure initiates pulmonary production of IL‐6 and IL‐17A, which subsequently circulate to the BM and promote osteogenic differentiation. As the primary target of inhaled exposure, the lung develops a pro‐inflammatory microenvironment upon external stimuli,^[^ ^41^ ^]^ consequently triggering the production of inflammatory cytokines. This response is supported by a prior study demonstrating that formaldehyde—a major VOC component—elevates the pulmonary levels of pro‐inflammatory cytokines.^[^ ^62^ ^]^ Within the inflamed lung tissue, these cytokines are predominantly released by a consortium of immune cells.^[^ ^63^, ^64^ ^]^ Myeloid cells, such as Macros and dendritic cells, contribute to IL‐6 production at sites of tissue injury,^[^ ^63^ ^]^ while IL‐17A secretion is dominated by T‐cell subsets, especially lung‐resident γδ T cells during early inflammatory phases.^[^ ^64^ ^]^ Critically, the local pulmonary cytokines inevitably translocate into the vasculature as a result of harmful inhalant‐increased vascular permeability.^[^ ^65^ ^]^ Once in the bloodstream, these cytokines can travel to the BM where they bind to receptors on niche cells, directly activating intracellular pathways that drive osteogenic differentiation.^[^ ^33^, ^66^, ^67^ ^]^ While the proposed lung‐to‐BM axis is supported by integrated experimental and literature evidence, further validation using advanced approaches—such as cytokine tracing and tissue‐specific knockout models—will help fully elucidate the spatiotemporal dynamics of these cytokines.

Our study delineates T cells as a reliable biomarker for evaluating VOCs‐induced lung diseases, provides a novel insight into immunological mechanisms from in situ lung and distal BM hematopoiesis, and offers potential targets (i.e., IL‐6 and IL‐17A) for therapeutic intervention of VOCs‐associated obstructive lung diseases. However, there are also inherent limitations in our study. Although our work addresses the integrated immunotoxicity of real‐world VOC mixtures, the linkages between innate and adaptive cells remain an important direction for future research. Additionally, while we reveal how BM‐derived T cells contributed to pulmonary immune replenishment, further work is required to probe the crucial role of pulmonary hematopoiesis, which may be more sensitive to inhaled stimuli during early exposure. To pay more attention to the crucial role of IL‐6 and IL‐17A on osteoblast differentiation in the BM niche, we ignored its migration and secretion mechanisms, which need to be further explored in future studies. Moreover, component‐specific toxicity and structure–activity relationships warrant in‐depth investigation in future research, which will significantly enhance mechanistic insight and inform public health relevance.

## 4 Experimental Section

### Animal Experiment Protocol

Six‐week‐old male C57BL/6 mice were purchased from Beijing Vital River Laboratory Animal Technology Co., Ltd. These animals were maintained in a controlled environment void of pathogens (with a temperature of 20 ± 2 °C and a 12‐h light/dark cycle) and had free access to food and water. Experimental procedures were approved by the Animal Care and Use Committee of Qingdao University (QDU‐AEC‐2024543) and Hebei Medical University (IACUC‐Hebmu‐20170116), carried out under the standards for ethical animal usage. Following the 1‐week acclimatization period, the animals were randomly divided into two groups (*n* = 24), i.e., the CON group and the VOC group. The exposure methodology for VOCs was adapted from the previous work, which established a whole‐body inhalation model for VOCs exposure.^[^ ^10^ ^]^ Mice were exposed to VOC exhaust generated from the volatilization of decoration paint in a paint factory located in Shijiazhuang, China. To ensure that the mice in the CON group were exposed to air void of particulate matter and VOCs, their breathing air was passed through high‐efficiency particulate air (HEPA) filters as well as filters containing activated carbon, which is efficient in adsorbing VOCs. In contrast, mice in the VOC group breathed the air filtered only by HEPA to remove particulate matter, allowing them to be exposed to the higher VOCs present in the chamber.

To assess the duration of exposure, the CON and VOCs‐exposed mice were further randomized into two separate subgroups (*n* = 12). One subgroup from each group was exposed for a 4‐week duration, while the other subgroup endured an 8‐week exposure period. Throughout the exposure periods, mice were kept in the CON or VOC chambers for 8 h day^−1^ (d), 7 d week^−1^ for a consecutive 4 or 8‐week duration of the experiment. Real‐time VOC concentrations within the chambers were monitored using the Personal Exposure Kit (PEK‐Standard 4 G, Sapiens Environmental Technology Co., Ltd., China), ensuring consistent and controlled exposure conditions. At the predetermined endpoints of the exposure period, the mice were anesthetized to detect lung function and to harvest the Bronchoalveolar Lavage Fluid (BALF), blood, and tissue samples for the subsequent assessment.

### Preparation of Single‐Cell Suspensions

To enable analysis of flow cytometry, single‐cell suspensions were prepared from BALF, lung, BM, and PB. For the preparation of BALF single‐cell suspension, 800 µL of cold phosphate‐buffered saline (PBS; Meilunbio, China) solution was instilled into the lungs via a tracheal cannula, and cells were aspirated. The collected cell suspension was then centrifuged at 600 × *g* for 5 min at 4 °C to pellet the cells. Lung tissues were isolated to prepare a pulmonary single‐cell suspension by a combination of mechanical disruption and enzymatic digestion, as described previously.^[^ ^68^ ^]^ The left lung lobe was cut into cubic pieces of ≈1 mm^3^ and incubated with collagenase I (1 mg mL^−1^, Solarbio, China) and deoxyribonuclease I (DNase I, 0.1 mg mL^−1^, Solarbio, China) in a gas bath constant‐temperature oscillator (Changzhou Gaode Instrument Manufacturing Co., Ltd., China) at 37 °C for 90 min. Subsequently, the cells were passed through a 70‐µm cell strainer (Biosharp, China), and the red blood cells (RBCs) were lysed using pharm RBC lysis buffer (Servicebio, China). Following lysis, the suspension was centrifuged at 400 × *g* for 10 min at 4 °C, and the resulting pellet was resuspended to obtain a single‐cell suspension of lung tissue. To prepare BM single‐cell suspension, the tibia and femur were flushed with Dulbecco's modified Eagle medium (DMEM; HyClone, USA) and then filtered with a 40‐µm cell strainer (Biosharp, China). The RBCs were removed from the BM suspension by the same method used for the lung cells. To prepare the single‐cell suspension of PB, 10 µL of the anticoagulated sample was diluted in 500 µL of PBS and centrifuged at 400 × *g* at 4 °C for 5 min. RBCs were removed from the resulting supernatant using the same method as for the lung cells, further obtaining a single‐cell suspension of circulating blood. The number of cells in each suspension was counted using an RWD‐C100‐SE automatic cell counter (RWD Life Science Co., Ltd., China). Subsequently, these suspensions were processed for the flow cytometry assay.

### Flow Cytometry Assay

For the determination of immune cells, single cells (≈5 × 10^6^ cells per sample) were harvested from the alveoli, lung, BM, and PB. These cells were then resuspended in 50 µL of cell buffer, which consisted of PBS supplemented with 1% fetal bovine serum (FBS, Gibco, USA), and incubated with antibodies from an immune panel for 30 min at room temperature. For the analysis of hematopoietic cells, single cells from the lung or BM were stained with antibodies from a hematopoietic panel using the above procedures as described for the immune cells. In addition, BM single‐cell suspension was stained with a mix of antibodies to identify BM niche cells. Details of the antibodies used for the above three multiple‐staining panels are provided in Table S17 (Supporting Information). Following antibody staining, the cells were re‐suspended in 500 µL of cell buffer and analyzed using a multi‐fluorescence flow cytometer (Beckman Coulter, USA). The gating of different populations of immune and hematopoietic cells, as well as BM niche cells, was performed using FlowJo software (TreeStar), based on the specific phenotypic characteristics outlined in Tables S18–S21 (Supporting Information). The original data were subsequently analyzed using R (version 4.2.2) and GraphPad Prism (version 8.0.1) for further statistical and graphical presentations.

### Immunofluorescence Assay

To conduct the immunofluorescence assay, mouse femur and lung tissues were first treated following a standard protocol. Briefly, mouse femur specimens were fixed in 4% paraformaldehyde (PFA, Servicebio, China) for 24 h, embedded in optimal cutting temperature (OCT) compound, and sectioned at a thickness of ≈10 µm. Moreover, the expressions of specific receptors in mouse lungs were assessed using an immunofluorescence assay. Mouse lung samples from different groups were gently extracted, washed with PBS, and immediately immobilized in 4% PFA for 24 h. After fixation, the lung tissues underwent a series of pretreatment steps, including dehydration with graded alcohol, embedding in paraffin, and sectioning to a thickness of ≈5 µm.

The sections of mouse femurs were subsequently incubated with Lineage, CD127, and Ki‐67 primary antibodies (all from Servicebio, China) at 4 °C overnight. After washing, the sections were incubated with a horse radish peroxidase (HRP)‐conjugated secondary antibody (Servicebio, China) for 50 min. Similarly, mouse lung sections were incubated with primary antibodies against CXCR3 (Bosterbio, China), CCR5 (Servicebio, China), and CD8 (Servicebio, China) at 4 °C overnight, followed by the application of secondary antibodies. To counterstain the nuclei, 4',6‐diamidino‐2‐phenylindole (DAPI; Servicebio, China) was used. After staining, the slides were sealed with anti‐fluorescence quenching tablets and mounted in gelvatol for subsequent confocal analysis. The samples were photographed using a fluorescence microscope (Olympus, Japan) and analyzed with CaseViewer software (version 2.4.0). Finally, the quantification was performed in a blinded manner to ensure unbiased results.

### Histopathological Observation

To conduct a histopathological examination, the sections of mouse thymus tissues were prepared following a protocol analogous to that employed for the lung samples in the *Immunofluorescence Assay*. Upon processing, thymus and lung sections were stained with hematoxylin and eosin (H&E, Servicebio, China) to visualize cortical thickness of the thymus and histopathological lesions in the lung, respectively. Additionally, lung sections were also stained with periodic acid‐schiff (PAS, Bosterbio, China) to detect goblet cells and Masson's trichrome (Servicebio, China) to assess collagen fibers. In addition to the above stains, sections of mouse femur were subjected to Goldner trichrome (Bosterbio, China) staining to evaluate mineralization, TRAP (Servicebio, China) staining for the identification of osteoclasts, and Oil Red O (Servicebio, China) staining to evaluate the adipogenic differentiation of MSCs. Histopathological alterations were observed and photographed using a standard light microscope (Olympus, Japan). The quantification of the stained areas was conducted employing Image J (version 1.80) software in a blinded manner, ensuring objective and unbiased analysis of the histological changes.

### Immunohistochemical Assay

Paraffin‐embedded thymus and femur tissues were sectioned at a thickness of ≈4 µm. These sections were subsequently processed through a series of steps, including de‐paraffinization, re‐hydration, antigen retrieval, and serum blocking to prepare the tissue for subsequent antibody incubation. Specifically, thymus sections were incubated with the primary antibodies targeting CD3 and CD8 (both from Servicebio, China) at 4 °C overnight. Subsequently, the sections were thoroughly washed to remove any unbound primary antibodies and then incubated with the HRP‐conjugated secondary antibody for 50 min. In parallel, femur sections were incubated with primary antibodies against IL‐7 (Servicebio, China) and CXCL12 (Servicebio, China) at 4 °C overnight, followed by incubation with the corresponding secondary antibody. After visualizing with diaminobenzidine (DAB; Servicebio, China), the slides were observed under a standard light microscope (Olympus, Japan). The stained slides were then evaluated, and the intensity of the immune reactivity was quantified using Image J (version 1.80) software.

### Enzyme‐Linked Immunosorbent Assay (ELISA)

To detect cytokine levels in the alveoli, BALF was subjected to centrifugation at 600 × *g* for 5 min at 4 °C. The resulting cell‐free supernatant was carefully collected for subsequent analysis. Mouse lung tissue was homogenized and lysed with a radio immunoprecipitation assay lysis buffer (Beyotime, China) to obtain the total cellular protein. After centrifuging at 5000 × *g* at 4 °C for 10 min, the supernatant was collected for tissue cytokine analysis. For serum samples, blood collections were rested for 2 h at room temperature to allow clotting, after which they were centrifuged at 800 × *g* for 10 min to separate the serum from the cellular fraction. To obtain the BM supernatant, DMEM flushed from the tibia and femur was centrifuged at 600 × *g* for 5 min to ensure the isolation of the cell‐free supernatant. Following protein quantification utilizing the BCA assay kit (Epizyme, USA), the prepared samples were subjected to ELISA assay. The concentrations of CXCL9, CXCL10, and CXCL11 in the serum were quantified using specific ELISA kits (Solarbio, China). CXCL10 level was also measured in both the alveoli and lung tissues. Additionally, ELISA kits (Solarbio, China) were used to determine the levels of interleukins, including IL‐6, IL‐10, IL‐17A, IL‐18, and IL‐13 in the BM, serum, and lung samples. Absorbance readings at 450 nm were obtained using a multifunction microplate reader (Thermo Fisher Scientific, USA) to assess cytokine levels. The results were expressed in picograms per milliliter (pg mL^−1^), calculated based on the prepared standard curve for each cytokine.

### Micro‐CT Analysis

To conduct micro‐CT analysis, femurs fixed with 4% PFA were scanned using the Quantum GX2 µCT Imaging System (PerkinElmer, USA). The scanning protocol was established with a voltage of 80 kV, a current of 100 µA, and a resolution of 9 µm per pixel to ensure high‐quality imaging. Following data acquisition, a comprehensive 3D reconstruction and visualization process was performed using CT Analyser software (version 1.11.0.0) and Dataviewer software (version 1.4.3), as previously described.^[^ ^69^ ^]^ Trabecular bone analysis was focused on a predetermined region, which encompassed 5% of the femoral length proximal to 0.1 mm above the distal femoral growth plate. This specific region was chosen to ensure that the analysis was conducted in a growth plate proximal area, which is known to be a critical zone for bone development and remodeling. Within this region, the following parameters were calculated: BV/TV, trabecular number (Tb.N), and Tt.Ar.

### Isolation and Initial Culture of Mouse BMSCs

Mouse BMSCs were isolated from male C57BL/6 mice, following a protocol previously described.^[^ ^70^ ^]^ Briefly, 6‐week‐old mice were humanely euthanized, and their femurs were carefully collected on ice following a 20‐min decontamination period in 75% ethanol. To harvest the BMSCs, the femurs were flushed with DMEM using syringes. The obtained cells were then filtered with a 40‐µm cell strainer and centrifuged at 100 × *g* for 5 min. The resulting cell pellet was resuspended in fresh DMEM medium, which was supplemented with 10% FBS and 1% penicillin (100 U mL^−1^)/streptomycin (0.1 mg mL^−1^) (Solarbio, China). The cells were seeded into culture dishes (10‐cm diameter) and placed in a humidified environment maintained at 5% CO_2_ and 37 °C. After 24 h, non‐adherent cells were removed, and the adherent cells were washed twice with PBS. These cells were then cultured with fresh medium, with the medium being replaced every 3 d. Following 7 d of culture, the mouse BMSCs reached ≈70% confluence, at which point they were ready for sub‐culturing. The sub‐culturing process involved washing the cells twice with PBS, incubating them with 0.25% trypsin (Solarbio, China) for 2 min to dissociate the cells, neutralizing the trypsin activity with isochoric complete medium, centrifuging the cells (100 × *g*, 5 min), and finally inoculating them into a new culture dish.

### Ex Vivo Assay for Osteogenic Differentiation of mouse BMSCs

Mouse BMSCs were seeded into 12‐well plates with a density of 2 × 10^5^ cells per well and cultured in basal culture medium (i.e., DMEM) at 37 °C. After 24 h incubation, the medium was replaced with osteogenic‐inducing medium (OIM), which was composed of DMEM supplemented with 10% FBS, 1% penicillin (100 U mL^−1^)/streptomycin (0.1 mg mL^−1^), 0.1 µm dexamethasone (Macklin, China), 5 mm β‐glycerolphosphate (Saitong, China), and 50 µg mL^−1^ ascorbic acid (Aladdin, China). The OIM was changed every 3 d to maintain the osteogenic culture conditions. For the ex vivo assay, 10% FBS in the OIM was replaced with 10% serum obtained from VOCs‐exposed or control mice. For the identification of crucial regulatory cytokines, 3 µg mL^−1^ of anti‐IL‐6 (Biocell, China) and/or anti‐IL‐17A (Biocell, China) antibodies were added to the culture medium containing mouse serum from VOCs‐exposed group, either individually or in combination. After 24 h of incubation, the mouse BMSCs were harvested for subsequent assessment.

### ALP Staining and Activity Assay

To perform ALP staining, mouse BMSCs were first carefully washed with PBS and then fixed in 4% PFA for 15 min. Following fixation, the cells were again washed with PBS and stained with the ALP staining solution (Beyotime, China) at 37 °C for 30 min. After the staining period, the cells were washed twice with PBS and observed under a microscope (EVOS™ XL Core, Thermo Fisher Scientific, USA) for visualization of ALP staining. For the ALP activity assay, the BMSCs were gently washed with PBS, lysed with 0.5% Triton X‐100 on ice, and then centrifuged at 600 × *g* for 30 min at 4 °C. Total protein concentration in the supernatant was determined using a BCA protein assay kit (Epizyme, USA) to ensure accurate quantification of ALP activity. Subsequently, the ALP activity was measured at 37 °C using an ALP assay kit (Beyotime, China) according to the manufacturer's instructions. The activity of ALP was normalized to the total protein content of each sample and expressed in U/gprot.

### Quantitative Polymerase Chain Reaction (qPCR) assay

Total Ribonucleic Acid (RNA) was extracted from homogenized mouse lungs and BM single‐cell suspensions. The concentration and purity of the extracted RNA were determined using a NanoDrop®ND‐1000 spectrophotometer (Thermo Fisher Scientific, USA). The extracted RNA was then reverse‐transcribed to complementary Deoxyribonucleic Acid (cDNA) using a reverse transcription kit (Takara, Japan) according to the manufacturer's instructions. Subsequently, *q*PCR assays were performed on the cDNA samples using SYBR Green MasterMix (Yeasen, China) and quantified in a QuantStudio 7 Flex Real‐Time PCR System (Thermo Fisher Scientific, USA), as previously reported.^[^ ^71^ ^]^ Primer sequences for target or reference genes were listed in Table S22 (Supporting Information). The relative levels of test genes were calculated by the 2^−ΔΔCt^ method, with *β‐actin* as the housekeeping gene for normalization.^[^ ^72^ ^]^

### Population Cohort Study

A population‐based cohort study was conducted in Shijiazhuang, Hebei Province, China. The study aimed to assess the impact of indoor VOCs exposure on respiratory health in a cohort of individuals who were free from a history of lung cancer and exhibited no clinical symptoms indicative of lung cancer. Participants aged 25 to 70 years were enrolled. Participants aged 25–70 years were enrolled and categorized into two groups based on whether home decoration had been completed for more than one year, including 21 in the CON group and 11 in the exposure group. All participants provided informed consent prior to enrollment. The study protocol was reviewed and approved by the Ethics Committee of Hebei Medical University (Approval number: 2016021).

For the data collection, demographic information was gathered through structured face‐to‐face interviews utilizing a standardized questionnaire. This information included age, gender, educational level, lifestyle habits such as cigarette smoking and alcohol consumption, duration of in‐home exposure, and a history of chronic diseases (hypertension, diabetes mellitus, heart disease, and respiratory disease). For the exposure assessments, the environmental concentrations of VOCs were measured via a handheld VOC gas detector (NEO MP180 PLUS, mPower Electronics, China) in each sample. A noninvasive pulmonary function test was conducted utilizing a portable pulmonary function testing machine (Beijing M&B Electronic Instruments Co., Ltd., China). Lung function indicators included FIV_05_, FVC, FIV_1_/FVC, MIF_50_, PIF, FEV_1_, FEV_1_/FVC, EXtime, peak expiratory flow (FEF), maximum mid‐expiratory flow (MMF), mid‐expiratory flow at 25% (MEF_25_), mid‐expiratory flow at 50% (MEF_50_), and mid‐expiratory flow at 75% (MEF_75_). Fresh blood samples from all participants were processed under standardized conditions and analyzed for complete blood cell counts using a Beckman Coulter HmX hematology analyzer (Beckman Coulter, USA). Additionally, plasma samples were then collected to detect the CXCL10, IL‐6, and IL‐17A levels by ELISA.

### Statistical Analysis

All experimental data were expressed as mean ± standard error of the mean (SEM) with a minimum of three analytical duplicates per group. Statistical analysis was performed using GraphPad Prism (version 8.0.1) and R software (version 4.1.2). The data between CON and VOC groups were compared using a two‐tailed unpaired *t*‐test or one‐way analysis of variance (ANOVA) following testing for normal distribution and homogeneity of variance. For high‐dimensional datasets, false discovery rate (FDR) correction was applied to adjust for multiple comparisons. A comprehensive correlational analysis was conducted using Pearson's correlation coefficient to investigate both the magnitude and directionality of linear relationships between continuous variables of interest. Following z‐score standardization of continuous variables, multiple linear regression was employed to assess associations between target variables. To ensure model parsimony and avoid overfitting, analyses were adjusted for key demographic and lifestyle covariates, such as age, BMI, smoking status, and alcohol drinking status. However, exposure estimates lacking significant confounding effects, e.g., ventilation, home size, and renovation history (data not shown), were excluded from final models. To visually represent these results, GraphPad Prism (version 8.0.1), R software (version 4.1.2), and bioinformatics (available at http://www.bioinformatics.com.cn/) as graphing tools were utilized.

A mediation analysis was employed using the R package “mediation” to delineate the direct and indirect impacts of VOCs on lung risks (assessed by lung function indicators), with a specific focus on the potential mediating role of LYM. This examination provided three key estimates: (I) total effect, representing the overall association between VOCs and lung risks; (II) direct effect, providing a focused elucidation of the independent relationship between VOC levels and lung function indicators; and (III) indirect effect, unveiling the mediating role of LYM in VOCs‐induced lung risks. The proportion of mediation by LYM was quantified using the following formula: mediation proportion = (indirect effect/total effect) × 100%. Significant differences were considered in all tests when *p* was less than 0.05 or 0.01.

## Conflict of Interest

The authors declare no conflict of interest.

## Author Contributions

H.Y. and J.Z. are co‐first authors and contributed equally to this work. H.Y. and J.Z. conceived and designed the study, analyzed the data, and wrote the manuscript. H.Y. and J.Z. designed and conducted all experiments, unless otherwise indicated. Q.L. recruited populations and collected the samples. D.L. checked the whole manuscript for potential grammar errors and typos. R.P. analyzed the histology and immunohistochemistry data. G.M. performed the cohort study and conducted data analysis on mediation effects. Y.C. and Z.K. performed and analyzed the *q*PCR experiments. G.Q. and R.Z. provided technical help for multi‐fluorescence flow cytometry and whole‐body inhalation exposure model. X.J. and Y.Z. discussed the experiments and results.

## Supporting information



Supporting Information

## Data Availability

The data that support the findings of this study are available from the corresponding author upon reasonable request.
