# Supplementary material for: Interior Decorative VOCs Elevate T Cell‐Mediated Obstructive Lung Disease Risks via Osteogenesis‐Driven Lymphoid‐Biased Hematopoiesis
Source: Adv Sci (Weinh). 2025 Nov 5;13(4):e12663. doi: 10.1002/advs.202512663 (PMC12822434; doi:10.1002/advs.202512663)
Supplement: Supplementary file 1 — Supporting Information [file ADVS-13-e12663-s001.docx]

**Supplementary Materials for**

**Interior Decorative VOCs Elevate T Cell-Mediated Obstructive Lung Disease Risks via Osteogenesis-Driven Lymphoid-Biased Hematopoiesis**

Hongyan Yu,^1†^ Jingxu Zhang,^1†^ Qingping Liu,^2^ Duxing Li,^3^ Ruonan Pan,^1^ Gan Miao,^1^ Yidi Chen,^1^ Zhe Kou,^1^ Guangbo Qu,^4,5,6^ Rong Zhang,^2^* Xiaoting Jin,^1^* Yuxin Zheng ^1^

^1^ Department of Occupational and Environmental Health, School of Public Health, Qingdao University, Qingdao, Shandong, 266071, China

^2^ Department of Toxicology, School of Public Health, Hebei Medical University, Shijiazhuang, Hebei, 050017, China

^3^ ZaoZhuang Center for Disease Control and Prevention, Zaozhuang, Shandong, 277101, China

^4^ State Key Laboratory of Environmental Chemistry and Ecotoxicology, Research Center for Eco-Environmental Sciences, Chinese Academy of Sciences, Beijing, 100085, China

^5^ College of Resources and Environment, University of Chinese Academy of Sciences (UCAS), Beijing, 101408, China

^6^ School of Environment, Hangzhou Institute for Advanced Study, UCAS, Hangzhou, Zhejiang, 310058, China

†These authors contributed equally to this work.

*Corresponding authors. Emails: rongzhang@hebmu.edu.cn (R. Zhang), xtjin@qdu.edu.cn (X. Jin).

**This file includes:**

Supplementary Text

Figures S1 to S10

Tables S1 to S22

References

Supplementary Text

**Concentrations of Interior Decoration Volatile Organic Compounds (VOCs)**

The concentrations of indoor VOC were measured according to the methods exactly as described previously. We monitored VOC concentration in the chambers weekly using Personal Exposure Kit (PEK-Standard 4G, Sapiens Environmental Technology Co., Ltd) until the endpoint of exposure (8-week).

**Body Weight and Organ Coefficient for Mice**

Employing an accurate electronic balance, the body weight (g) of mice was recorded once before grouping and then recorded weekly after the onset of exposure. At the endpoint of exposure, mice from each group were sacrificed in a humane manner and the main organs, including the heart, lung, liver, and spleen were rapidly excised. We next recorded the harvested organ weights (g) of mice. Histological examination of the major organs was conducted. The organ coefficient was then calculated using the following formula: Organ coefficient (%) = organ weight/body weight × 100.

**Pulmonary Function Testing**

To assess pulmonary function, six mice from each group were anesthetized with pentobarbital sodium to ensure a calm and cooperative state for testing. The FinePointe™ Pulmonary Function Testing System (DSI Instrument Co., Ltd, ST. PAUL) was utilized to measure respiratory parameters. As previously described ^[1]^, this testing involved carefully exposing the trachea, making a small incision, inserting a calibrated cannula, and securing it in place with sutures. The mice were subsequently connected to the ventilator component of the pulmonary function system. The ventilator was adjusted to synchronize with the tracheal cannula, ensuring a consistent pressure throughout the testing process. The ventilator was operated at a respiratory rate of 140 breaths per min (BPM) to ensure adequate ventilation. Following a stabilization period to allow the mice to adapt to the breathing circuit, the pulmonary function parameters, including forced vital capacity (FVC), forced expiratory volume in one second (FEV_1_), the peak expiratory flow rates at 25% and 75% of FVC (FEF_25_ and FEF_75_), and the maximal mid-expiratory flow (MMEF), were automatically monitored and recorded by the system.

Supplementary Figure

**
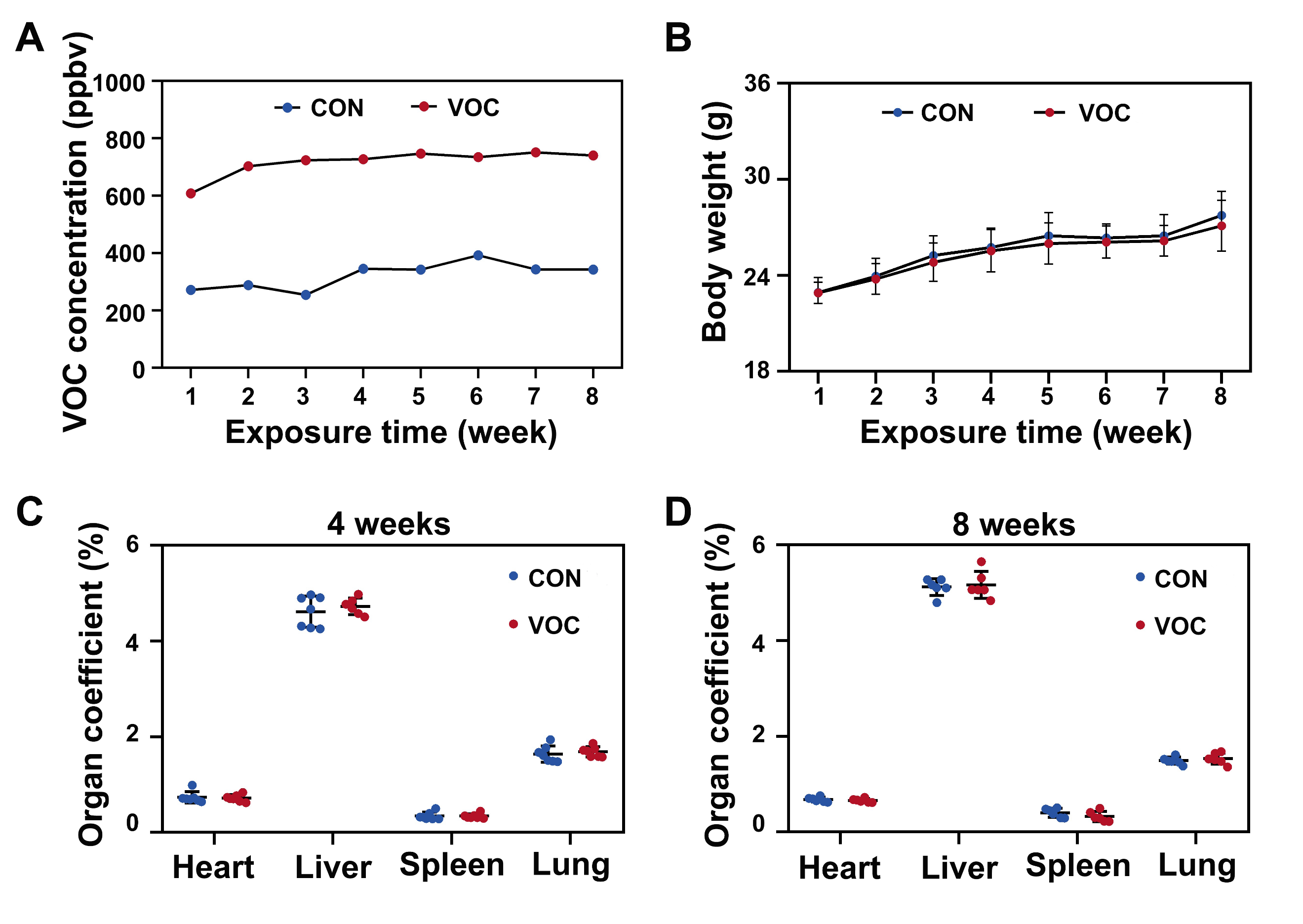
**

**Fig. S1. Assessment of mice treated with or without VOCs exposure.** (**A**) Concentrations of VOCs during the exposure period. (**B**) Body weights of mice during the exposure period. (**C-D**) Organ coefficients of the heart, liver, spleen, and lung after 4-week or 8-week treatment. All values were mean ± SEM.


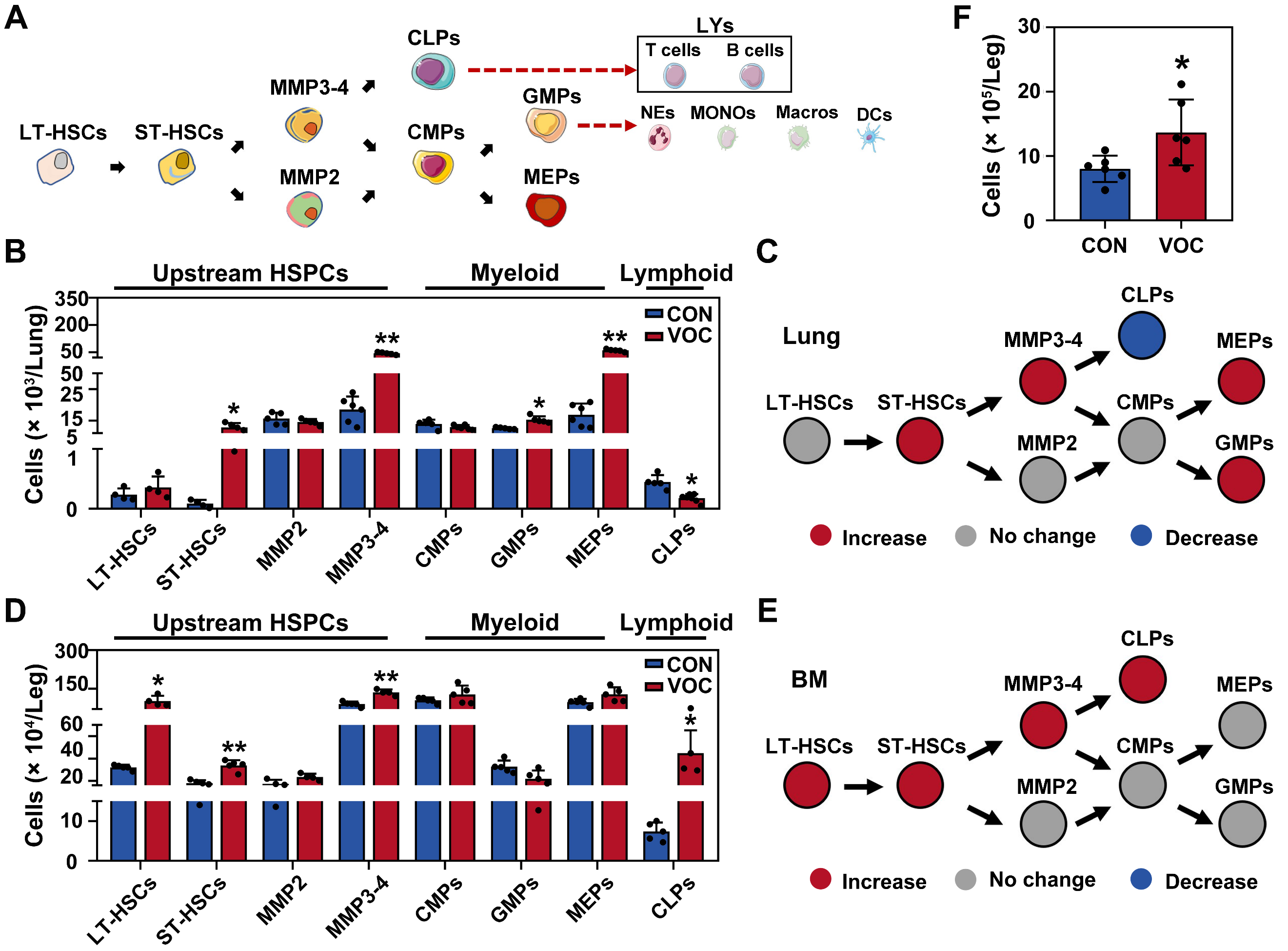


**Fig. S2. Effects of 4-week VOCs exposure on hematopoiesis of mice.** (**A**) Schematic model for hematopoietic process and generation of immune cells. (**B**) Total numbers of hematopoietic cells in the lung of mice after 4-week exposure in CON or VOC chambers. (**C**) Schematic diagram for the pulmonary hematopoiesis upon 4-week VOCs exposure. (**D**) Quantification of hematopoietic cell numbers in the BM of mice after 4-week exposure in CON or VOC chambers. (**E**) Schematic diagram for the BM hematopoiesis upon 4-week VOCs exposure. (**F**) Quantitative analysis of T cells in the BM of mice upon 8-week VOCs exposure. ******p* < 0.05, and *******p* < 0.01 versus the corresponding control group, using Student’s t-test. All values were mean ± SEM.


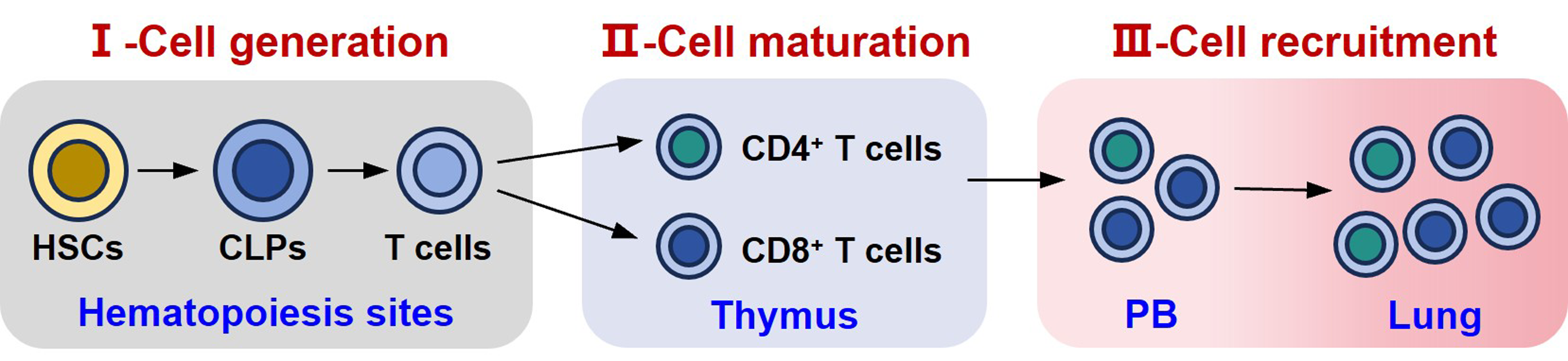


**Fig. S3. Three critical stages involved in the replenishment of T cells to the lung tissues.**


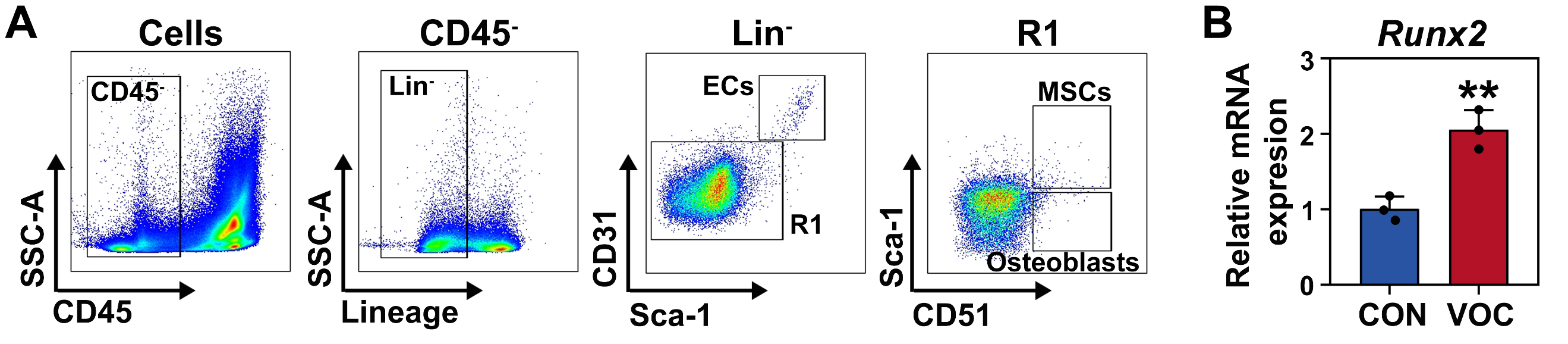


**Fig. S4. Assessment of osteogenic differentiation in mice treated with or without VOCs exposure.** (**A**) Representative flow cytometric dot plots of MSCs and osteoblasts in BM niche of mice. (**B**) Relative expression of *Runx2* in mouse femur. *******p* < 0.01 versus the corresponding control group, using Student’s t-test. All values were mean ± SEM.


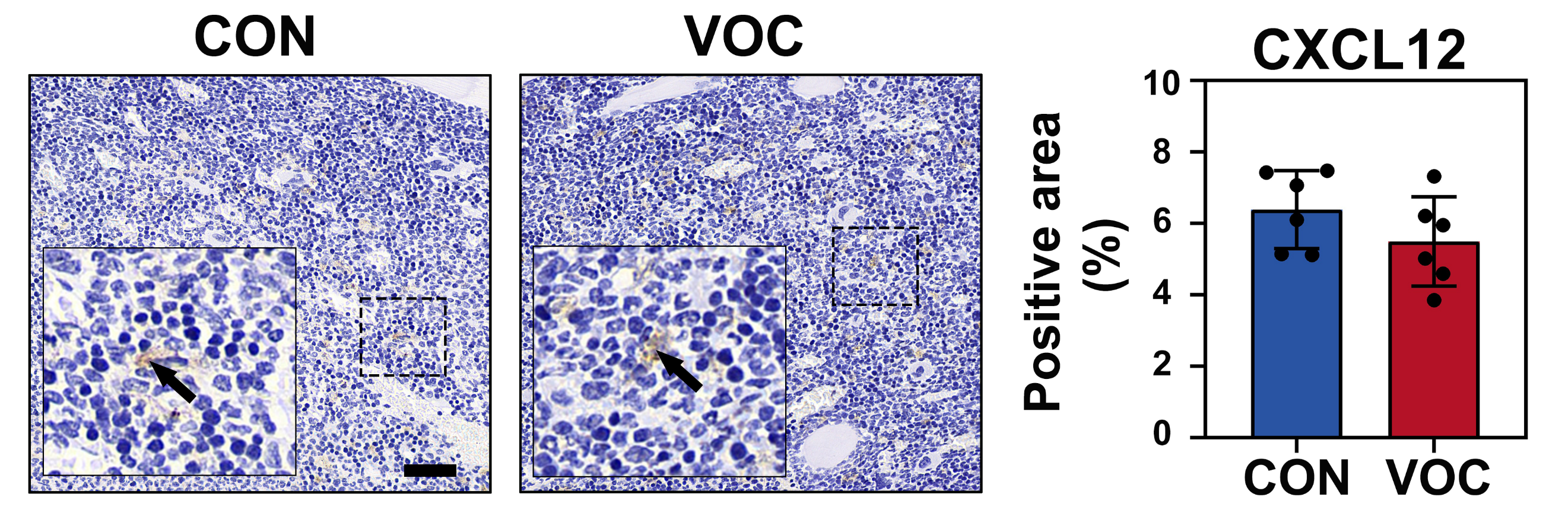


**Fig. S5. Representative images of immunohistochemical staining for CXCL12 and corresponding quantification of positive area.** Black arrows indicate positive expression positive area. Scale bar: 40 μm. All values were mean ± SEM.


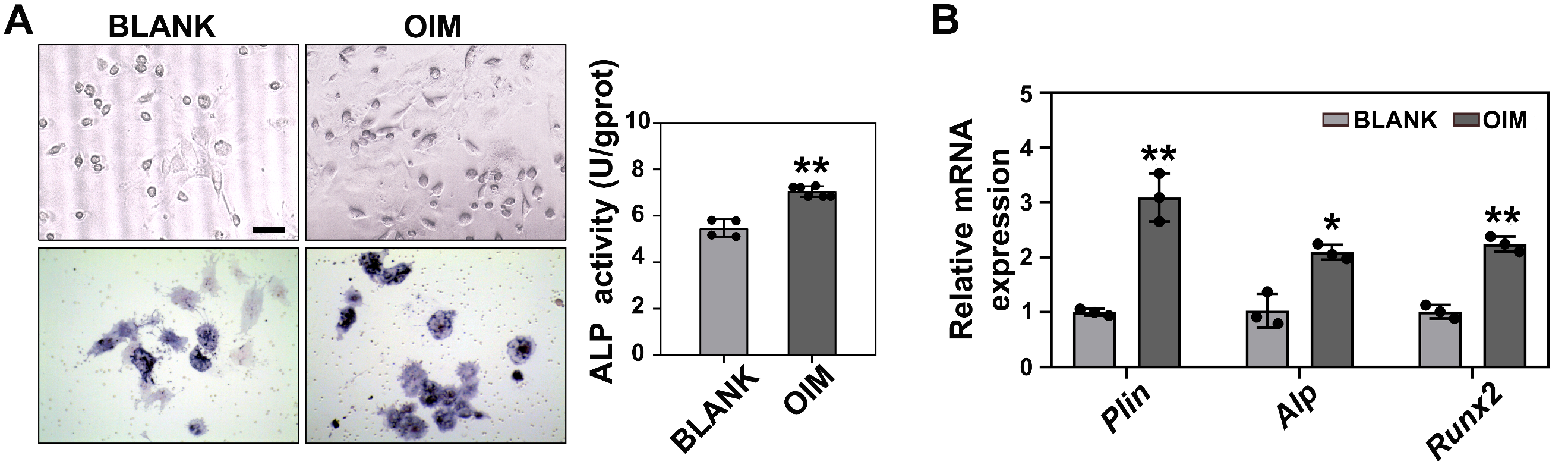


**Fig. S6. Effects of osteogenic-inducing medium on mouse bone marrow mesenchymal stem cells (BMSCs).** (**A**) Osteogenesis was determined by ALP staining and ALP activity assays. Scale bar: 400 μm. (**B**) Relative mRNA levels of *Plin*, *Alp*, and *Runx2* in mouse BMSCs. ******p* < 0.05, and *******p* < 0.01 versus the BLANK group, using Student’s t-test. All values were mean ± SEM.


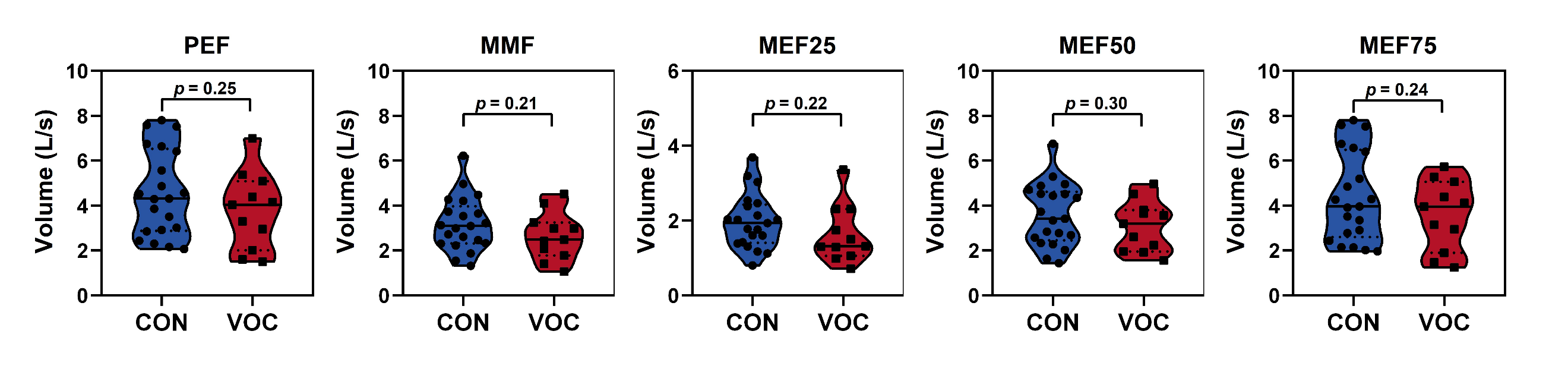


**Fig. S7. Results of lung function parameters in humans.** Data were presented as mean ± SD or median (IQR). Student’s t-test and rank sum test were used to examine the subgroup differences.


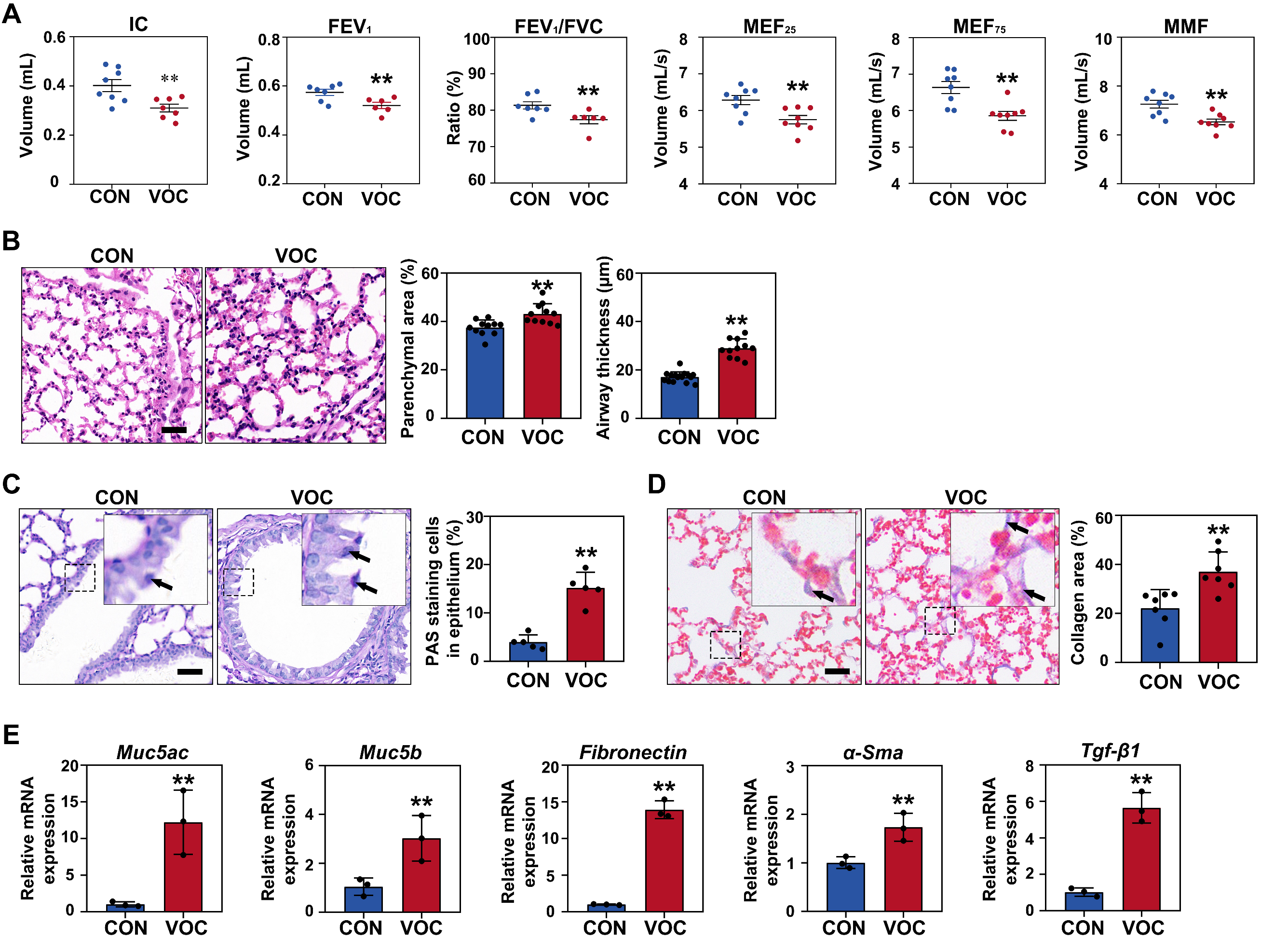


**Fig. S8. Detrimental impacts of VOCs on lung function and structure in a mouse model.** (**A**) Results of lung function parameters in mice at 8 weeks. (**B**) Representative images of lung sections stained with H&E and quantitative assessment of the pulmonary parenchymal area and airway wall thickness. Scale bar: 40 μm. (**C**) Representative images of lung sections stained with PAS, along with a detailed quantitative analysis. Scale bar: 40 μm. (**D**) Representative images of lung sections stained with Masson’s trichrome and the percentage of collagen area. Scale bar: 40 μm. (**E**) *Muc5ac*, *Muc5b*, *Fibronectin*, *α-Sma*, and *Tgf-β1* levels were detected by *q*PCR. *******p* < 0.01 versus the corresponding control group, using Student’s t-test. All values were mean ± SEM.


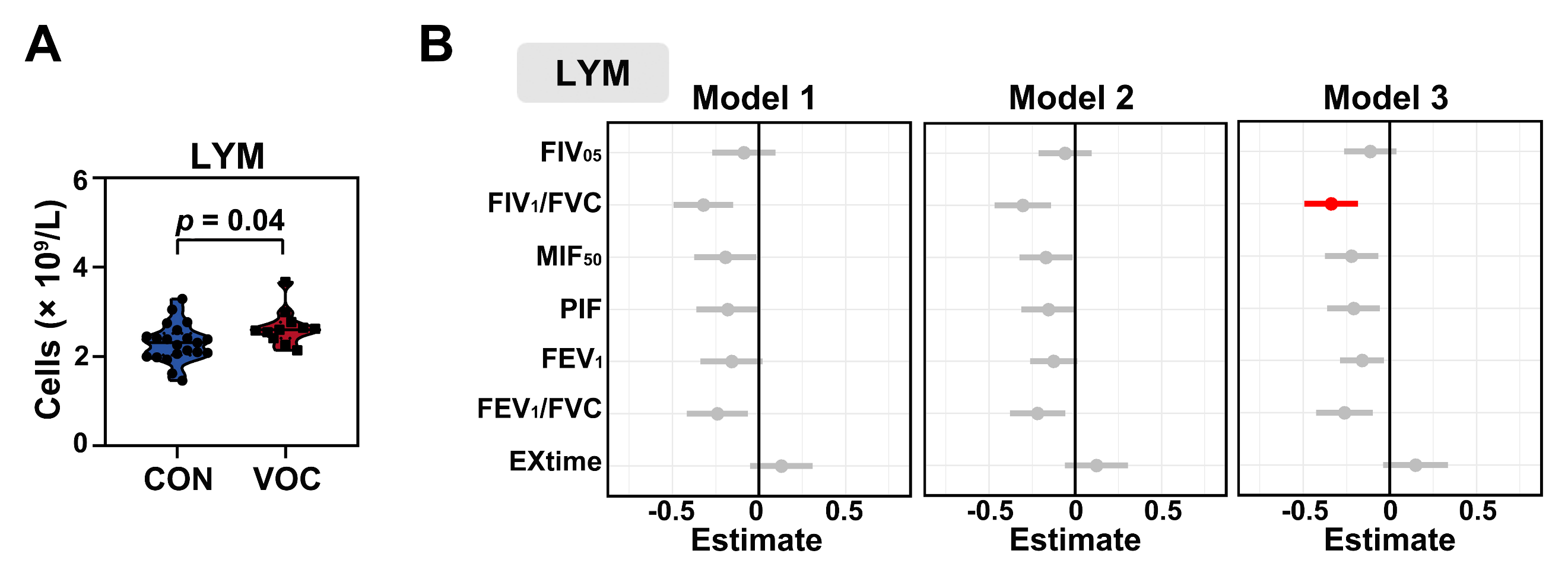


**Fig. S9. Quantification of LYM and its correlation with VOC concentrations.** (**A**) Quantitative assessment of the number of LYM in the PB. (**B**) Forest plots indicating the association between LYM numbers and lung function parameters. Model 1: Without adjustment. Model 2: Adjusted for age. Model 3: Model 2 with additional adjustment for BMI, smoking status, and alcohol drinking status.


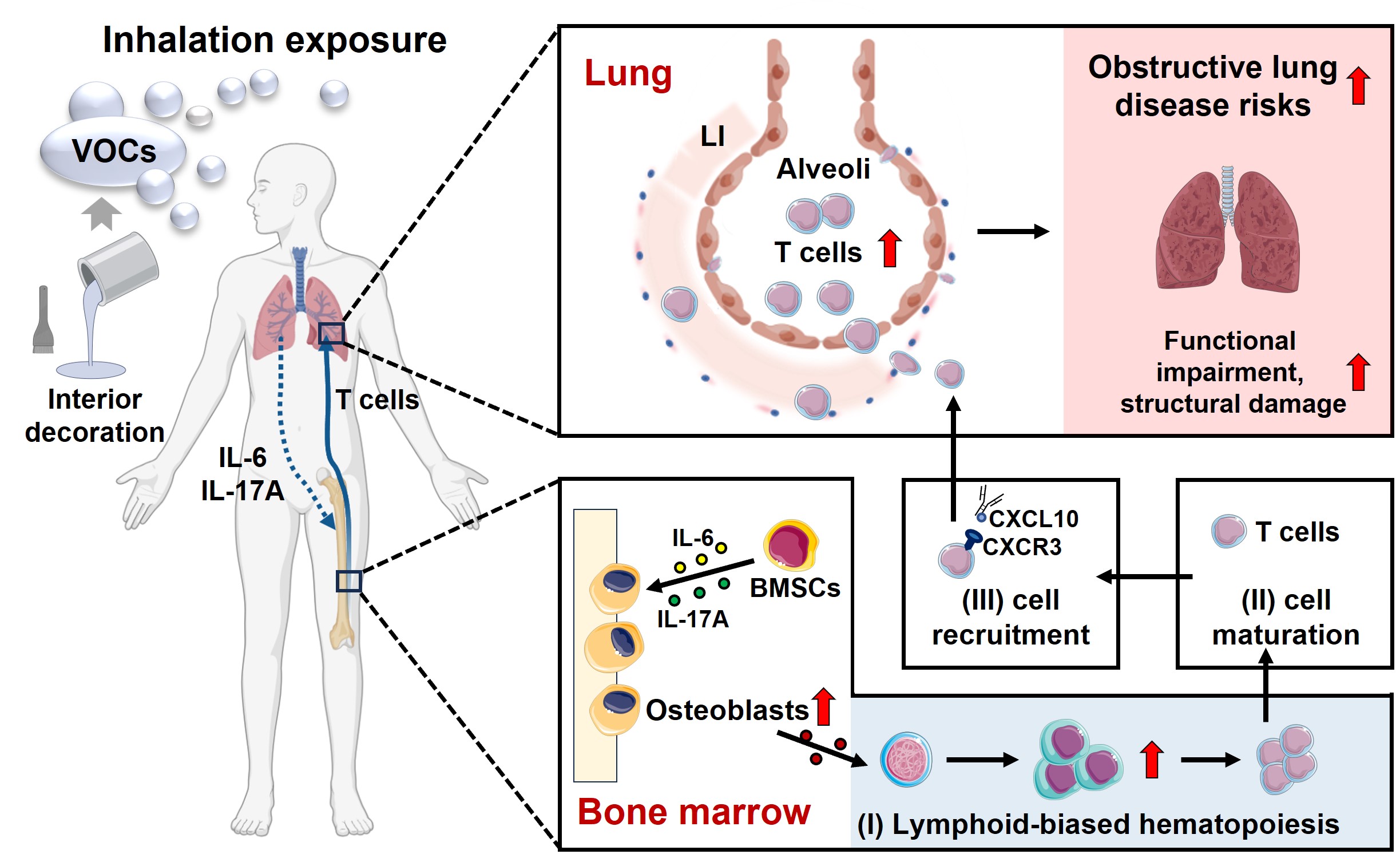


**Fig. S10. A schematic depiction of the characteristics and mechanisms of immune cell compositions in the lung, as well as the detrimental respiratory impacts induced by interior decorative VOCs exposure.**

Supplementary Table

**Table S1. Summary data for immune cells in the alveoli at 8 weeks. Related to Figure 1.**

| Cells | CON  (Mean ± SEM) | VOC  (Mean ± SEM) |
| --- | --- | --- |
| CD4^+^ T | 3858.0 ± 127.1 | 6746.0 ± 503.3 |
| CD8^+^ T | 764.7 ± 19.7 | 1175.7 ± 60.4 |
| B | 5765.0 ± 548.2 | 5597.7 ± 1166.3 |
| NEs | 2983.5 ± 61.6 | 2965.7 ± 512.1 |
| Macros | 11674.0 ± 593.4 | 11692.5 ± 1972.4 |

**Table S2. Summary data for immune cells in the LI at 8 weeks. Related to Figure 1.**

| Cells | CON  (Mean ± SEM) | VOC  (Mean ± SEM) |
| --- | --- | --- |
| CD4^+^ T | 423843.5 ± 8566.7 | 419150.4 ± 4895.6 |
| CD8^+^ T | 563774.9 ± 8885.2 | 724199.2 ± 18321.7 |
| B | 931498.1 ± 23716.3 | 1038399.2 ± 12853.7 |
| NEs | 778829.9 ± 113231.0 | 565094.9 ± 54075.3 |
| Macros | 523933.4 ± 10227.7 | 619818.9 ± 4761.4 |
| DCs | 37337.0 ± 4529.2 | 41116.2 ± 5435.6 |
| MONOs | 90722.0 ±3189.6 | 85755.8 ± 8003.4 |

**Table S3. Summary data for pulmonary hematopoiesis cells at 4 weeks. Related to Figure S2.**

| Cells | CON  (Mean ± SEM) | VOC  (Mean ± SEM) |
| --- | --- | --- |
| LT-HSCs | 236.3 ± 41.7 | 285.2 ± 64.8 |
| ST-HSCs | 57.3 ± 24.6 | 2797.5 ± 406.2 |
| MPP2 | 5644.7 ± 543.3 | 4563.3 ± 264.3 |
| MPP3-4 | 8522.6 ± 926.4 | 43787.2 ± 1892.9 |
| CMPs | 4663.7 ± 210.2 | 2346.3 ± 86.9 |
| GMPs | 2615.7 ± 82.8 | 5827.6 ± 307.6 |
| MEPs | 8904.4 ± 1165.3 | 59822.9 ± 1991.4 |
| CLPs | 449.0 ± 33.3 | 178.1 ± 12.3 |

**Table S4. Summary data for pulmonary hematopoiesis cells at 8 weeks. Related to Figure 2.**

| Cells | CON  (Mean ± SEM) | VOC  (Mean ± SEM) |
| --- | --- | --- |
| LT-HSCs | 2452.8 ± 264.3 | 605.7 ± 43.7 |
| ST-HSCs | 17255.7 ± 1474.9 | 8893.7 ± 602.9 |
| MPP2 | 13476.6 ± 945.8 | 9303.7 ± 379.4 |
| MPP3-4 | 106780.7 ± 9023.1 | 127404.4 ± 6193.6 |
| CMPs | 11117.7 ± 1002.9 | 6704.7 ± 227.0 |
| GMPs | 14843.2 ± 850.0 | 13473.4 ± 689.1 |
| MEPs | 153701.8 ± 13474.4 | 194950.1 ± 13183.9 |
| CLPs | 916.8 ± 85.4 | 647.0 ± 69.2 |

**Table S5. Summary data for BM hematopoiesis cells at 4 weeks. Related to Figure S2.**

| Cells | CON  (Mean ± SEM) | VOC  (Mean ± SEM) |
| --- | --- | --- |
| LT-HSCs | 32160.3 ± 644.8 | 101692.0 ± 8308.7 |
| ST-HSCs | 17902.9 ± 786.3 | 33814.8 ± 1373.1 |
| MPP2 | 17400.8 ± 1421.6 | 23526.8 ± 1162.3 |
| MPP3-4 | 90115.5 ± 2864.6 | 135148.4 ± 4709.1 |
| CMPs | 104889.7 ± 3084.8 | 127159.1 ± 9979.3 |
| GMPs | 32664.0 ± 1596.4 | 21960.3 ± 2838.2 |
| MEPs | 96753.0 ± 3817.8 | 127830.0 ± 7864.1 |
| CLPs | 7388.8 ± 652.4 | 25090.9 ± 8363.6 |

**Table S6. Summary data for BM hematopoiesis cells at 8 weeks. Related to Figure 2.**

| Cells | CON  (Mean ± SEM) | VOC  (Mean ± SEM) |
| --- | --- | --- |
| LT-HSCs | 17094.8 ± 454.1 | 22584.5 ± 2378.6 |
| ST-HSCs | 6940.1 ± 500.3 | 11013.9 ± 609.2 |
| MPP2 | 4050.6 ± 487.8 | 4667.9 ± 1006.6 |
| MPP3-4 | 99044.8 ± 6332.4 | 116805.6 ± 4129.7 |
| CMPs | 160268.4 ± 10894.8 | 160739.5 ± 5089.8 |
| GMPs | 31730.7 ± 1756.2 | 39404.0 ± 3950.9 |
| MEPs | 51837.5 ± 3852.5 | 33793.3 ± 4024.1 |
| CLPs | 3058.3 ± 150.4 | 7411.7 ± 533.3 |

**Table S7. Summary data for immune cells in the peripheral blood at 8 weeks. Related to Figure 3.**

| Cells | CON  (Mean ± SEM) | VOC  (Mean ± SEM) |
| --- | --- | --- |
| CD4^+^ T | 7463.9 ± 1137.7 | 8322.6 ± 1319.3 |
| CD8^+^ T | 7805.5 ± 758.89 | 8283.0 ± 807.8 |

**Table S8. Summary data for BM niche cells. Related to Figure 4.**

| Cells | CON  (Mean ± SEM) | VOC  (Mean ± SEM) |
| --- | --- | --- |
| MSCs | 12876.5 ± 599.0 | 24597.7 ± 889.1 |
| Osteoblasts | 15708.9 ± 508.8 | 25320.5 ± 879.1 |

**Table S9. Baseline characteristics among the study participants.**

| Variables | CON (n = 21) | VOC (n = 11) |
| --- | --- | --- |
| Age (mean ± SD, years) | 40.5 ± 13.3 | 54.4 ± 13.6 |
| Gender (n, %) |  |  |
| Males | 7 (33.3) | 5 (45.5) |
| Females | 14 (66.7) | 6 (54.5) |
| Smoking status (n, %) |  |  |
| Never smokers | 19 (90.5) | 10 (90.9) |
| Former smokers | 0 (0) | 1 (9.1) |
| Current smokers | 2 (9.5) | 0 (0) |
| Alcohol drinking status (n, %) |  |  |
| Never drinkers | 12 (57.2) | 4 (36.4) |
| Occasional drinkers | 7 (33.3) | 7 (63.6) |
| Frequent drinkers | 2 (9.5) | 0 (0) |
| In-home time, n (%) |  |  |
| Less than 6 hours | 0 (0) | 2 (18.2) |
| 7-11 hours | 14 (66.7) | 3 (27.2) |
| 12-17 hours | 4 (19.0) | 2 (18.2) |
| More than 18 hours | 3 (14.3) | 4 (36.4) |
| Education, n (%) |  |  |
| Middle school or below | 2 (9.5) | 4 (36.4) |
| High school | 2 (9.5) | 2 (18.2) |
| Bachelor | 9 (42.9) | 3 (27.2) |
| Master or beyond | 8 (38.1) | 2 (18.2) |
| Past medical history, n (%) |  |  |
| Hypertension | 4 (19.1) | 0 (0) |
| Diabetes mellitus | 2 (9.5) | 0 (0) |
| Heart diseases | 0 (0) | 0 (0) |
| Respiratory diseases | 4 (19.0) | 1 (9.1) |

**Table S10. Summary data for lung function parameters of humans. Related to Figure 6.**

| Variable | CON (n = 21) | VOC (n = 11) | *p*-value |
| --- | --- | --- | --- |
| FIV_05_ (L, median (IQR)) | 0.8 (0.5) | 0.1 (0.4) | < 0.01^b^ |
| FIV_1_/FVC (%, median (IQR)) | 73.8 (29.2) | 24.7 (53.8) | < 0.01^b^ |
| MIF_50_ (L/s, median (IQR)) | 2.7 (1.3) | 1.8 (1.9) | 0.02^b^ |
| PIF (L/s, median (IQR)) | 3.1 (1.3) | 1.8 (1.7) | 0.02^b^ |
| FEV_1_ (L, median (IQR)) | 2.0 (1.0) | 0.6 (1.3) | < 0.01^b^ |
| FEV_1_/FVC (%, mean ± SD) | 75.8 ± 20.8 | 36.6 ± 33.1 | < 0.01^a^ |
| EXtime (s, mean ± SD) | 1.9 ± 0.6 | 3.3 ± 1.7 | 0.02^a^ |
| PEF (L/s, mean ± SD) | 4.6 ± 1.9 | 3.8 ± 1.7 | 0.24^a^ |
| MMF (L/s, mean ± SD) | 3.2 ± 1.2 | 2.6 ± 1.1 | 0.20^a^ |
| MEF_25_ (L/s, mean ± SD) | 2.0 ± 0.7 | 1.6 ± 0.8 | 0.23^a^ |
| MEF_50_ (L/s, mean ± SD) | 3.6 ± 1.4 | 3.1 ± 1.1 | 0.27^a^ |
| MEF_75_ (L/s, median (IQR)) | 4.0 (3.6) | 4.0 (2.3) | 0.36^b^ |

Data were presented as mean ± standard deviation or median (IQR). ^a^ Student’s t-test was used to examine the subgroup differences. ^b^ Rank sum test was used to examine the subgroup differences.

**Table S11. Correlation analysis of LYM proportions with lung function parameters. Related to Figure 6.**

| Variable | Model 1 | | Model 2 | | Model 3 | |
| --- | --- | --- | --- | --- | --- | --- |
|  | β (95% CI) | *p*-value | β (95% CI) | *p*-value | β (95% CI) | *p*-value |
| FIV_05_ | -0.60 (-0.89, -0.31) | < 0.01 | -0.45 (-0.74, -0.16) | < 0.01 | -0.39 (-0.68, -0.10) | 0.02 |
| FIV_1_/FVC | -0.59 (-0.86, -0.32) | < 0.01 | -0.52 (-0.85, -0.19) | < 0.01 | -0.46 (-0.79, -0.13) | 0.01 |
| MIF_50_ | -0.56 (-0.87, -0.25) | < 0.01 | -0.42 (-0.73, -0.11) | 0.01 | -0.38 (-0.67, -0.09) | 0.02 |
| PIF | -0.55 (-0.84, -0.26) | < 0.01 | -0.40 (-0.71, -0.09) | 0.02 | -0.35 (-0.64, -0.06) | 0.03 |
| FEV_1_ | -0.60 (-0.87, -0.33) | < 0.01 | -0.39 (-0.64, -0.14) | < 0.01 | -0.33 (-0.58, -0.08) | 0.02 |
| FEV_1_/FVC | -0.54 (-0.85, -0.23) | < 0.01 | -0.42 (-0.73, -0.11) | 0.02 | -0.37 (-0.70, -0.04) | 0.04 |
| Extime | 0.33 (-0.02, 0.68) | 0.07 | 0.32 (-0.05, 0.69) | 0.11 | 0.27 (-0.12, 0.66) | 0.19 |

Model 1: Without adjustment. Model 2: Adjusted for age. Model 3: Model 2 with additional adjustment for BMI, smoking status, and alcohol drinking status.

**Table S12. Correlation analysis of LYM numbers with lung function parameters. Related to Figure S9.**

| Variable | Model 1 | | Model 2 | | Model 3 | |
| --- | --- | --- | --- | --- | --- | --- |
|  | β (95% CI) | *p*-value | β (95% CI) | *p*-value | β (95% CI) | *p*-value |
| FIV_05_ | -0.09 (-0.44, 0.26) | 0.63 | -0.06 (-0.35, 0.23) | 0.7 | -0.11 (-0.42, 0.20) | 0.48 |
| FIV_1_/FVC | -0.32 (-0.67, 0.03) | 0.07 | -0.31 (-0.62, 0) | 0.07 | -0.34 (-0.63, -0.05) | 0.05 |
| MIF_50_ | -0.20 (-0.55, 0.15) | 0.29 | -0.17 (-0.48, 0.14) | 0.28 | -0.24 (-0.53, 0.05) | 0.13 |
| PIF | -0.18 (-0.53, 0.17) | 0.32 | -0.16 (-0.45, 0.13) | 0.32 | -0.22 (-0.51, 0.07) | 0.15 |
| FEV_1_ | -0.16 (-0.51, 0.19) | 0.39 | -0.13 (-0.38, 0.12) | 0.36 | -0.17 (-0.42, 0.08) | 0.21 |
| FEV_1_/FVC | -0.24 (-0.59, 0.11) | 0.18 | -0.22 (-0.53, 0.09) | 0.18 | -0.25 (-0.56, 0.06) | 0.13 |
| Extime | 0.13 (-0.22, 0.48) | 0.48 | 0.12 (-0.23, 0.47) | 0.51 | 0.14 (-0.23, 0.51) | 0.46 |

Model 1: Without adjustment. Model 2: Adjusted for age. Model 3: Model 2 with additional adjustment for BMI, smoking status, and alcohol drinking status.

**Table S13. Correlation analysis of VOC concentrations with lung function parameters. Related to Figure 6.**

| Variable | Model 1 | | Model 2 | | Model 3 | |
| --- | --- | --- | --- | --- | --- | --- |
|  | β (95% CI) | *p*-value | β (95% CI) | *p*-value | β (95% CI) | *p*-value |
| FIV_05_ | -0.21 (-0.56, 0.14) | 0.24 | -0.22 (-0.49, 0.05) | 0.16 | -0.12 (-0.43, 0.19) | 0.50 |
| FIV_1_/FVC | -0.48 (-0.79, -0.17) | < 0.01 | -0.48 (-0.77, -0.19) | < 0.01 | -0.38 (-0.71, -0.05) | 0.04 |
| MIF_50_ | -0.27 (-0.60, 0.06) | 0.14 | -0.27 (-0.56, 0.02) | 0.09 | -0.23 (-0.56, 0.10) | 0.17 |
| PIF | -0.26 (-0.61, 0.09) | 0.15 | -0.27 (-0.56, 0.02) | 0.09 | -0.21 (-0.52, 0.10) | 0.21 |
| FEV_1_ | -0.23 (-0.58, 0.12) | 0.21 | -0.23 (-0.48, 0.02) | 0.09 | -0.14 (-0.41, 0.13) | 0.34 |
| FEV_1_/FVC | -0.42 (-0.75, -0.09) | 0.02 | -0.42 (-0.71, -0.13) | < 0.01 | -0.39 (-0.70, -0.08) | 0.03 |
| Extime | 0.53 (0.22, 0.84) | < 0.01 | 0.53 (0.22, 0.84) | < 0.01 | 0.53 (0.18, 0.88) | < 0.01 |

Model 1: Without adjustment. Model 2: Adjusted for age. Model 3: Model 2 with additional adjustment for BMI, smoking status, and alcohol drinking status.

**Table S14. Mediation analysis of LYM proportions in the overall reduction in FIV_1_/FVC induced by VOCs. Related to Figure 6.**

|  | Estimate | 95% CI | *p*-value |
| --- | --- | --- | --- |
| Total effect | -0.48 | (-0.90, -0.25) | < 0.01 |
| Mediation effect | -0.14 | (-0.30, -0.02) | 0.01 |
| Direct effect | -0.34 | (-0.79, -0.10) | < 0.01 |
| Proportion mediated (%) | 29.26 | (4.28, 73.00) | 0.01 |

Adjusting variables: age, BMI, smoking status, and alcohol drinking status.

**Table S15. Mediation analysis of LYM proportions in the overall reduction in FEV_1_/FVC induced by VOCs. Related to Figure 6.**

|  | Estimate | 95% CI | *p*-value |
| --- | --- | --- | --- |
| Total effect | -0.42 | (-0.75, -0.19) | < 0.01 |
| Mediation effect | -0.11 | (-0.25, 0.03) | 0.10 |
| Direct effect | -0.32 | (-0.71, -0.06) | 0.01 |
| Proportion mediated (%) | 25.05 | (-6.16, 74.00) | 0.10 |

Adjusting variables: age, BMI, smoking status, and alcohol drinking status.

**Table S16. Concentrations of the top 10 high-abundance VOC components.**

| VOCs species | CON (ppbv) | VOC (ppbv) |
| --- | --- | --- |
| Formaldehyde | 7.41 | 9.68 |
| Acetaldehyde | 7.05 | 7.52 |
| Ethylene | 1.64 | 3.23 |
| n-Butene | 2.71 | 3.16 |
| n-Nonane | 1.38 | 2.62 |
| Dichloromethane | 1.89 | 2.56 |
| Methacrolein | 0.77 | 2.42 |
| Ethane | 1.59 | 2.20 |
| n-Octane | 1.35 | 2.01 |
| Propane | 0.98 | 1.61 |

**Table S17. Antibodies used in the optimized multicolor immunofluorescence panel.**

| Immune panel | | | | Hematopoietic panel | | | | BM niche panel | | |  |
| --- | --- | --- | --- | --- | --- | --- | --- | --- | --- | --- | --- |
| Antibody | fluorochrome | | Clone | Antibody | fluorochrome | Clone | Antibody | | fluorochrome | Clone | |
| CD45 | APC/cy7 | 30-F11 | | 7AAD | Percp/cy5.5 |  | CD45 | | APC/cy7 | 30-F11 | |
| CD4 | PE | GK1.5 | | Lineage | Percp/cy5.5 |  | 7AAD | | Percp/cy5.5 |  | |
| CD8a | PE/Cy5 | 53-6.7 | | CD45 | APC/cy7 | 30-F11 | Lineage | | Percp/cy5.5 |  | |
| CD19 | BV480 | 1D3 | | Sca-1 | BV605 | D7 | Sca-1 | | BV605 | D7 | |
| CD11b | FITC | M1/70 | | CD117 | PE | 2B8 | CD31 | | APC | W18222B | |
| CD11c | eFlur^TM^450 | N418 | | CD150 | APC | mShad150 | CD51 | | PE | RMV-7 | |
| F4/80 | PE/Cy7 | BM8 | | CD48 | BV421 | HM48-1 |  | |  |  | |
| Ly-6C | PE/D594 | HK1.4 | | CD34 | FITC | RAM34 |  | |  |  | |
| Ly-6G | APC | 1A8 | | CD16/32 | BV510 | 2.4G2 |  | |  |  | |
|  |  |  | | CD127 | PE/Cy7 | ATR34 |  | |  |  |  |

**Table S18. Phenotypes of immune cells in the alveoli.**

|  | CD45 | CD11b | CD11c | CD4 | CD8 | CD19 | F4/80 | Ly6G | Ly6C |
| --- | --- | --- | --- | --- | --- | --- | --- | --- | --- |
| CD4^+^ T | + |  | - | + |  |  |  |  |  |
| CD8^+^ T | + |  | - |  | + |  |  |  |  |
| B | + |  | - |  |  | + |  |  |  |
| NEs | + | + |  |  |  |  |  | + | + |
| Macros | + |  | + |  |  |  | + | - | - |

**Table S19. Phenotypes of immune cells in the lung interstitium and BM.**

|  | CD45 | CD11b | CD11c | CD4 | CD8 | CD19 | Ly6G | F4/80 |
| --- | --- | --- | --- | --- | --- | --- | --- | --- |
| CD4^+^ T | + | - | - | + |  |  |  |  |
| CD8^+^ T | + | - | - |  | + |  |  |  |
| B | + | - | - |  |  | + |  |  |
| NEs | + | + |  |  |  |  | + |  |
| DCs | + |  | + |  |  |  |  | - |
| MONOs | + |  | - |  |  |  |  | - |
| Macros | + |  | + |  |  |  |  | + |

**Table S20. Phenotypes of BM niche cells.**

|  | Lineage | CD45 | Sca-1 | CD31 | CD51 |
| --- | --- | --- | --- | --- | --- |
| ECs | - | - | + | + |  |
| MSCs | - | - | + | - | + |
| Osteoblasts | - | - | - | - | + |

**Table S21. Phenotypes of hematopoietic cells in the lung and BM.**

|  | Lineage | CD45 | Sca-1 | CD117 | CD150 | CD48 | CD34 | CD16/32 | CD127 |
| --- | --- | --- | --- | --- | --- | --- | --- | --- | --- |
| LSK | - | + | + | + |  |  |  |  |  |
| LK | - | + | - | + |  |  |  |  |  |
| LT-HSCs | - | + | + | + | + | - |  |  |  |
| ST-HSCs | - | + | + | + | - | - |  |  |  |
| MPP2 | - | + | + | + | + | + |  |  |  |
| MPP3-4 | - | + | + | + | - | + |  |  |  |
| CMPs | - | + | - | + |  |  | + | - |  |
| GMPs | - | + | - | + |  |  |  | + |  |
| MEPs | - | + | - | + |  |  | - | - |  |
| CLPs | - | + | low | low |  |  |  |  | + |

**Table S22. Primer sequences for the target genes.**

| **Gene** | **Primer Sequences (5’ to 3’)** |
| --- | --- |
| *Plin* | Forward: CTGTGTGCAATGCCTATGAGA |
|  | Reverse: CTGGAGGGTATTGAAGAGCCG |
| *Alp* | Forward: CGCTGCCCGAATCCTTA |
|  | Reverse: GCTGATATGCGATGTCCTT |
| *Runx2* | Forward: AACGATCTGAGATTTGTGGGC |
|  | Reverse: CCTGCGTGGGATTTCTTGGTT |
| *Muc5ac* | Forward: CAGCAGTTGGCATTCAGGGTCAG |
|  | Reverse: AAGAGGAAGCGGCAGGTAGGTAG |
| *Muc5b* | Forward: TCAGCATCCGCCTAGTCCTCAC |
|  | Reverse: GGGAGTTGTGGGAGACCGTAGAG |
| *Fibronectin* | Forward: ACCAACAACAGCCACTCAGAAG |
|  | Reverse: GTAACCGACACGCAGCCATCC |
| *α-Sma* | Forward: CAACGCCTCCGCCATTCACTC |
|  | Reverse: CTGACCATAAGCACCGCCCAAC |
| *Tgf-β1* | Forward: TGGCTGTCCTGGAACTCACTCTG |
|  | Reverse: AGGCAAAGGCAAGCGAATCTCTG |
| *β-actin* | Forward: CCTCTATGCCAACACAGTGC |
|  | Reverse: ATACTCCTGCTTGCTGATCC |

References

1. Zhang Y, Liu Q, Ning J, Jiang T, Kang A, Li L, et al. The proteasome-dependent degradation of ALKBH5 regulates ECM deposition in PM(2.5) exposure-induced pulmonary fibrosis of mice. *J Hazard Mater*. 2022;432:128655.
